# Random Tagging Genotyping by Sequencing (rtGBS), an Unbiased Approach to Locate Restriction Enzyme Sites across the Target Genome

**DOI:** 10.1371/journal.pone.0143193

**Published:** 2015-12-03

**Authors:** Elena Hilario, Lorna Barron, Cecilia H. Deng, Paul M. Datson, Nihal De Silva, Marcus W. Davy, Roy D. Storey

**Affiliations:** 1 The New Zealand Institute for Plant & Food Research Ltd, Priv. Bag 92–169, Auckland, 1025, New Zealand; 2 The New Zealand Institute for Plant & Food Research Ltd, 412 No 1 Road RD 2, Te Puke, 3182, New Zealand; Nanjing Forestry University, CHINA

## Abstract

Genotyping by sequencing (GBS) is a restriction enzyme based targeted approach developed to reduce the genome complexity and discover genetic markers when *a priori* sequence information is unavailable. Sufficient coverage at each locus is essential to distinguish heterozygous from homozygous sites accurately. The number of GBS samples able to be pooled in one sequencing lane is limited by the number of restriction sites present in the genome and the read depth required at each site per sample for accurate calling of single-nucleotide polymorphisms. Loci bias was observed using a slight modification of the Elshire et al. method: some restriction enzyme sites were represented in higher proportions while others were poorly represented or absent. This bias could be due to the quality of genomic DNA, the endonuclease and ligase reaction efficiency, the distance between restriction sites, the preferential amplification of small library restriction fragments, or bias towards cluster formation of small amplicons during the sequencing process. To overcome these issues, we have developed a GBS method based on randomly tagging genomic DNA (rtGBS). By randomly landing on the genome, we can, with less bias, find restriction sites that are far apart, and undetected by the standard GBS (stdGBS) method. The study comprises two types of biological replicates: six different kiwifruit plants and two independent DNA extractions per plant; and three types of technical replicates: four samples of each DNA extraction, stdGBS vs. rtGBS methods, and two independent library amplifications, each sequenced in separate lanes. A statistically significant unbiased distribution of restriction fragment size by rtGBS showed that this method targeted 49% (39,145) of BamH I sites shared with the reference genome, compared to only 14% (11,513) by stdGBS.

## Introduction

The cost of high throughput DNA sequencing techniques has steadily decreased over time allowing genome sequence information to be obtained from a large number of organisms. Many applications require genotype information from multiple individuals within a population of interest; however it is generally too expensive to sequence fully all individuals, especially those with large genomes. Several methods have been developed to sequence a subset of the genome allowing genotypic information to be obtained from larger numbers of individuals. A targeted approach increases the sequencing coverage of select regions, reduces error rate and costs, allows sample multiplexing, and exploits the full capacity of current next generation sequencing platforms. Methods that rely on physical features of the genome do not require *a priori* sequence information and take advantage of the diversity of restriction enzyme (RE) sites, methylation patterns, and 3D genome organization. Reduced representation sequencing methods based on RE sites focus on the sequence heterogeneity around those sites across many individuals to extract useful variant information. To be effective, these methods need to accurately capture the genome sequence information in a reproducible way and should not be biased, especially if the technique is applied to association studies across sets of germplasm. By reducing the number of genomic regions to be sequenced, it is possible to increase the coverage of the targeted restriction sites or increase the number of individuals assessed in a population. Greater complexity reduction can be obtained by this approach and, in theory, will lead to higher sampling depth of the same genomic sites and reduce the amount of missing data. Genotyping by sequencing (GBS, [1]) restriction associated DNA (RAD) sequencing [2] and reduced representation libraries (RRL) are examples of strategies based on physical features [3]. GBS is based on relatively frequent restriction sites, preferentially not included in repeat rich regions of the genome, which generate smaller cloned fragments easily amplified by PCR, and allows sample multiplexing by incorporating a barcoded oligonucleotide in one of the adapters. The GBS method has been modified to incorporate a double digestion step and a Y-adapter to reduce the length of the restriction fragment [4], as well as an extended common adaptor oligonucleotide for selectively retrieving ApeK I restriction sites with a 3’-end modified common adaptor oligonucleotide where the ApeK I overhang’s redundant position (W (A or T)) is fixed to A, and two extra bases (X (any nucleotide), Y (C or T)) are extended beyond into the insert [5], which is a clever way of reducing the enormous RE site space produced by frequent RE cutters such as ApeK I.

The effect of the targeted fragment size on coverage has been documented in maize and rice [6], where the depth of sequencing coverage per site is biased towards smaller fragments. We have also noted an apparent bias on the representation of DNA sequences obtained using a slight modification [7] of the Elshire et al. method [1] (this study). A significant read coverage depth at each locus is required to accurately distinguish between heterozygous and homozygous sites. The read coverage depth is a function of the total number of single end reads deliverable by the sequencing instrument and the number of individuals multiplexed per lane. The selection of a plexity level is influenced by the haploid genome size and GC content. For an RE that cuts a large genome (>600 Mbp) frequently, the coverage per restriction site will be poor. A theoretical calculation sheet has been developed by the RAD sequencing team at the University of Edinburgh [8] to assist with RE selection. This is perhaps the most important step of any GBS or RAD sequencing experiment.

Some of the technical reasons why a biased restriction site representation is observed in GBS experiments are the quality of genomic DNA (gDNA), the RE and DNA ligase reaction efficiency, PCR bias towards the amplification of smaller restriction fragments during library amplification and at the cluster formation of the Illumina sequencing process. Another technical issue is the presence of numerous secondary metabolites which can co-purify with plant nucleic acids, inhibit downstream enzymatic reactions or interfere with the extraction resulting in very low yields of high molecular weight DNA [9]. To prepare any kind of reduced representation library (RAD, GBS or RRL), more than 100 ng of high quality gDNA is required. High throughput gDNA extraction methods required for GBS studies often yield low amounts of high molecular weight gDNA; the entire sample may have to be used to prepare the library. Among the alternative ways of obtaining large concentrations of DNA is random priming gDNA by PCR. Random priming of nucleic acids was first reported by Taylor et al. [10] to produce a DNA complement of RNA template. This technique is still used for preparing cDNA template for cloning, DNA probes for Southern or Northern blots, or for parallel sequencing [11]. Primer extension pre-amplification PCR (PEP-PCR) was developed by Zhang and collaborators in 1992 to amplify genomes from single cells with a 15-mer random oligonucleotide [12]. Later on, Grothues and colleagues [13] combined a PEP-PCR approach with a 9-15-mer random primer tagged to a constant 17 b 5’-head [14] to amplify fungal DNA in sufficient quantities detectable by agarose gel electrophoresis. A similar approach was developed to amplify a 180 kbp plasmid [15] and an improved PEP-PCR approach to whole genome amplification using a 15-mer random oligonucleotide [16].

Here we propose a modified version of the Elshire et al. [1] method where the starting material for preparing the library is amplified gDNA tagged with a random hexamer linked to the common GBS oligonucleotide, with an average length of 2 kbp, instead of intact gDNA. This approach has two advantages: it allows the production of large quantities of DNA for library preparation from only a few nanograms of intact gDNA and, by randomly landing on the genome, some amplicons contain RE sites flanking long RE fragment which would be difficult to capture by the standard GBS method. We evaluated stdGBS libraries prepared with intact gDNA prepared vs. rtGBS libraries from six kiwifruit (*Actinidia chinensis*) plants prepared in parallel. We describe the fragment size distribution, number of RE sites retrieved and variability between the two methods.

## Materials and Methods

### Plant selection and gDNA extraction

Six kiwifruit plants (KFA—KFF) were selected based on family relationship, representing five different genotypes (KFA and KFB are clones). Two total gDNA extractions were performed per plant using fully developed leaf tissue, except for KFE where young leaf buds were used. The gDNA extractions were done with the Qiagen DNeasy Plant Mini kit, using 100 mg leaf tissue ground with liquid nitrogen. DNA integrity was determined by loading ~ 100 ng gDNA per preparation on a 1% agarose gel. All gDNA preparations produced fragments >20 kbp. DNA purity was determined by absorbance ratios at 260/280 nm and 260/230 nm; all preparations had values above 1.7 and 1.9, respectively. The DNA was quantified by fluorometry (Qubit™ dsDNA HS Assay, Life Technologies). The average yield per extraction was 2.5 μg. One microgram of intact gDNA was aliquoted per well of a 1 mL 96-deep well plate (KFA to KFF, A1 to H6, dispensed by column), and concentrated by sodium acetate/ethanol. The DNA pellet was resuspended in 30 μL of 10 mM Tris-HCl pH 8 and the concentration was determined again by fluorometry. The average concentration was 28 ng/μL. One microlitre of this solution was transferred to equivalent locations of a 96-well PCR plate to prepare random tagged gDNA.

### Production of random tagged gDNA template for GBS library preparation

#### Primer extension pre-amplification, PEP-PCR

Each gDNA aliquot was randomly tagged by primer extension pre-amplification PCR (PEP-PCR) as follows: an average of 28 ng of gDNA was mixed with 1X AccuPrime™ High Fidelity Buffer I, 1 unit AccuPrime™ Taq DNA polymerase High Fidelity (Life Technologies) and 10 pmol of the GBS common adapter oligonucleotide (negative strand) linked to a random hexamer at the 3’-end (CTCGGCATTCCTGCTGAACCGCTCTTCCGATCTNNNNNN), to a final volume of 50 μL. The amplification reaction conditions were: 94°C 2 min, 1 cycle; (94°C 40 s, 30°C 2 min, ramp 0.1°C/s, 48°C 4 min, 68°C 1 min), 50 cycles; 68°C 7 min, 1 cycle.

#### Touchdown PCR, TD-PCR

The randomly tagged gDNA (rtgDNA) of each extraction aliquot was amplified as follows: One microlitre of rtgDNA was added to 19 μL of PCR reaction solution containing 0.6 M trehalose, 40 mM Tris-HCl pH 8, 20 mM KCl, 20 mM (NH_4_)_2_SO_4_, 10 μg BSA, 0.5 mM MgSO_4_, 0.2 mM dNTP, 10 pmol GBS common adapter negative strand oligonucleotide (without the random hexamer), and 0.25 unit Platinum^®^ Pfx DNA polymerase (Life Technologies). The touchdown PCR [17] amplification profile was carried out as follows: 94°C 2 min, 1 cycle; (94°C 30 s, TD 60–50°C 30 s ramp at maximum speed, 68°C 1 min), 20 cycles; (94°C 30 s, 50°C 30 s, 68°C 1 min), 10 cycles; 68°C 7 min. Two TD-PCR reactions were set up per sample. After amplification, each TD-PCR sample was pulled into one well, precipitated by sodium acetate/ethanol and resuspended in 30 μL of 10 mM Tris-HCl pH 8. This solution contained the template to prepare the rtGBS libraries. The average size of the random tagged DNA amplicons is 2 kbp. The content of each cell was transferred to its corresponding well (A7 to H12) in the 1 mL 96-deep well plate containing the intact gDNA. The average amount of rtgDNA was 372 ng.

### GBS library preparation and sequencing

The GBS library preparations were prepared according to Elshire et al. [1] with the following modifications [7]: One microgram of gDNA of each plant was used for the restriction digestion (stdGBS libraries only); we selected BamH I according to the criteria established by the GenePool group at the University of Edinburgh [8] for RAD sequencing protocols; the adapters were annealed according to Ko et al. [18]; the adapter ligation step undertaken after digestion, without drying out the DNA/adapter mixture; a high fidelity enzyme was used for amplification (AccuPrime™ Taq DNA polymerase High Fidelity, Life Technologies); the amplification of the library was done on each individual GBS library, then pooled and cleaned up before sequencing. The GBS adapters were designed by Deena Bioinformatics [19]. The stdGBS and rtGBS libraries were prepared in parallel since both types of DNA template were dispensed into the same 1 mL 96-deep well plate. We used 20 units of BamH I- HF with CutSmart reaction buffer (New England Biolabs) for each RE digestion. The GBS libraries were amplified twice (one for each Illumina HiSeq2000 lane, see Experiment design section below), on the same experiment, but in different PCR machines running simultaneously. The average library size was 396 and 374 bp for library pools sequenced in Lane 1 and Lane 2, respectively. The average insert size of the pooled libraries was: 267 and 245 bp, respectively, after subtracting the adaptors (129 bp). Single end (100 b) Illumina sequencing was performed on a HiSeq2000 instrument by the Australian Genome Research Facility (Melbourne, Australia). An average of 191,934,054 reads and 19.19 Gb were obtained per lane.

### Processing of Illumina raw sequence data, mapping to kiwifruit ‘Hongyang’ genome assembly and variant discovery

The quality of sequencing reads was checked using FastQC-0.11.2 [20]. Reads with an average Phred score higher than 20 in the first 80 cycles were kept for analysis. The filtered reads were separated by sample based on corresponding barcode using pyrad_v.2.01 [21]. Reads for each individual sample were then mapped to the draft genome of *A*. *chinensis* ‘Hongyang’ ([22], version AONS01000000) with bowtie2-2.2.4 [23] in “end-to-end very sensitive” mode. The alignment files were sorted and compressed into BAM format with samtools-0.1.18 [24].

Custom perl scripts were used to check the quality and post-process binary alignment map (bam) files, which were then used for extracting genomic coordinates of BamH I RE sites and read depth information (bed files, [25]). The Bioconductor package [26] GenomicRanges was used to find the intersection of observed bed coordinates for experimental samples versus the bed coordinates of the known population of BamH I RE sites and repeat regions in the kiwifruit ‘Hongyang’ genome.

The complemented set of ranges, i.e. the gaps between RE sites, were used to visualize the distribution of the sampled restriction enzyme fragments by stdGBS and rtGBS against the population of BamH I RE sites expected in the ‘Hongyang’ genome. The distribution of restriction fragments was exponential and was estimated by maximum likelihood [27].

### Experimental design and variation analysis

#### The data

The six kiwifruit plants selected for the study constitute six biological replicates. Two independent gDNA extractions were performed from each plant and sampled four times, giving eight laboratory replicates which formed a total of 48 experimental units for the study. The experimental units were each subjected to two different treatment methods (stdGBS and rtGBS) generating 96 libraries for sequencing. The two treatment levels (stdGBS and rtGBS) were therefore paired within each experimental unit. The 96 libraries were finally subjected to two independent library amplifications which were run on separate lanes of one Illumina HiSeq2000 flow cell. Any systematic differences between lanes and amplifications were therefore confounded in the experimental design and this is labelled as a single factor ‘Lane’ with two levels. The data structure therefore comprised 6 plants x 2 extracts x 4 samples x 2 methods x 2 lanes. It is noted here that the 48 experimental units constituted a nested structure: plant, extract (plant) and aliquot (plant, extract). Each nested factor should be ideally treated as a random effect. The method (stdGBS and rtGBS) and lane must be treated as fixed effects, noting that their interaction could be random. Any interactions of experimental unit factors with method may be of interest and should also be considered as a random effect. Initial analysis suggested that there were very small differences between extract and aliquot levels. Hence, for the purpose of this analysis these were collapsed to a single factor. The response variable of interest, *y*_*ijkm*_, was defined as the number of BamH I sites estimated from reads mapped on to kiwifruit ‘Hongyang’ genome that had a library prepared from plant *i*, sample (extract x aliquot) *j* and then subjected to method *k* and sequenced in lane *m*. The statistical analysis aimed to ascertain if rtGBS increased the number of BamH I sites compared to the stdGBS method, and to quantify the variability in BamH I site count in relation to experimental unit factors.

#### The model specification

We specified two alternative statistical models for the analysis. The negative binomial distribution is commonly used to model count data when over-dispersion is present and the Poisson approximation is not adequate, hence in the first model assumed,

$${\text{y}}_{\text{ijkm}}\sim \text{ NB}\left(\mu_{\text{ijkm}},\sigma^{2}{}_{\text{ijkm}}\right)$$ (1)

which can be fitted as a generalized linear mixed model (GLMM) with the random and fixed effect predictors described earlier. The GLMMs are complex and difficult to fit and so pseudo-likelihood methods are used for optimisation. The second model log transformed the counts data and assumed a normal distribution,

$$\text{log}({\text{y}}_{\text{ijkm})}\sim \text{ N}\left(\mu_{\text{ijkm}},\sigma^{2}{}_{\text{ijkm}}\right)$$ (2)

which can be fitted as the simpler linear mixed model (LMM). Given the distribution assumptions, the linear model with predictors of the response can be specified as:

$${\text{log}}_{\text{e}}(\mu_{\text{ijkm}}),E\left[{\text{log}}_{e}\left(y_{ijkm}\right)\right]\;=\mu \;+\;P_{i}+\;S_{j}+\;m_{k}+\;l_{m}+\;Pm_{ik}$$ (3)

where the plant *P*_*i*_ ~ *N*(0, *σ* ^2^_*P*_) and sample effect *S*_*j*_ ~ *N*(0, *σ* ^2^_*S*_) were both specified as random effects and captured the biological and laboratory variability respectively among the library experimental units. The method *m*_*k*_ and *l*_*m*_ in model (3) were considered fixed effects. Note the two terms on the left side of Eq (3) are the linear predictor for models (1) and (2) respectively.

The GLMM and LMM described as such were fitted to the data using the GLIMMIX and MIXED procedures in SAS^®^ statistical software (SAS Institute Inc. 2013).

## Results

### Effect of input gDNA for library preparation on read count and mapped BamH I RE sites

The amount of intact gDNA needed for preparing stdGBS libraries was almost twice that for rtGBS libraries (0.86 ± 0.10 μg and 0.37 ± 0.05 μg, respectively, Table 1).

**Table 1:** Data yield per treatment. Processed Illumina reads with Phred scores ≥20 are tabulated according to lane and treatment, and amount of gDNA used for each library preparation.

	Illumina million reads					
	std GBS			rtGBS		
Plant_DNA extraction_sample	genomic DNA (ng)	Lane 1	Lane 2	amplified rtgDNA (ng)	Lane 1	Lane 2
KFA_1_1	0.93	1.64	1.73	0.45	1.59	1.61
KFA_1_2	0.81	2.08	1.9	0.38	2.17	2
KFA_1_3	1.07	1.78	1.76	0.32	1.35	1.39
KFA_1_4	0.91	1.77	1.67	0.39	2.19	2.15
KFA_2_1	0.73	2.15	2.02	0.39	2.08	1.95
KFA_2_2	0.84	1.82	1.73	0.37	1.53	1.49
KFA_2_3	0.58	1.64	1.64	0.3	1.57	1.7
KFA_2_4	0.97	1.8	1.6	0.36	1.7	1.62
Average ± SD	0.85 ± 0.15	1.84 ± 0.19	1.76 ± 0.14	0.37 ± 0.05	1.77 ± 0.32	1.74 ± 0.27
KFB_1_1	0.77	1.7	1.8	0.41	2.09	2.01
KFB_1_2	0.82	1.9	1.85	0.38	1.6	1.61
KFB_1_3	0.77	1.95	1.85	0.32	2.06	2.05
KFB_1_4	0.86	2.03	1.99	0.33	1.69	1.64
KFB_2_1	0.64	2.44	2.31	0.32	1.6	1.67
KFB_2_2	0.83	2.2	2.14	0.35	1.86	1.83
KFB_2_3	0.9	1.64	1.71	0.29	1.84	1.77
KFB_2_4	0.77	1.93	1.81	0.36	1.54	1.62
Average ± SD	0.79 ± 0.08	1.97 ± 0.26	1.93 ± 0.20	0.34 ± 0.04	1.78 ± 0.21	1.77 ± 0.18
KFC_1_1	1.13	1.95	1.8	0.4	1.74	1.74
KFC_1_2	1	2.02	2.03	0.34	1.77	1.77
KFC_1_3	1.02	1.94	1.84	0.3	1.69	1.73
KFC_1_4	1.03	1.75	1.82	0.33	1.52	1.54
KFC_2_1	0.68	2.01	1.94	0.32	1.77	1.75
KFC_2_2	0.67	2.25	2.07	0.32	1.37	1.28
KFC_2_3	0.75	2.05	1.93	0.29	1.77	1.82
KFC_2_4	0.79	1.71	1.59	0.35	1.6	1.57
Average ± SD	0.88 ± 0.18	1.96 ± 0.17	1.88 ± 0.15	0.33 ± 0.04	1.65 ± 0.15	1.65 ± 0.18
KFD_1_1	0.84	1.77	0.26	0.38	2.03	1.92
KFD_1_2	0.85	1.76	0.27	0.39	1.29	1.15
KFD_1_3	0.79	1.52	0.22	0.31	1.41	1.42
KFD_1_4	0.84	1.71	0.26	0.34	0.92	0.87
KFD_2_1	0.78	1.85	0.28	0.35	1.85	1.8
KFD_2_2	0.86	1.8	0.28	0.34	1.83	1.76
KFD_2_3	0.8	1.72	0.25	0.34	1.27	1.28
KFD_2_4	0.84	1.58	0.23	0.33	1.46	1.44
Average ± SD	0.82 ± 0.03	1.71 ± 0.11	0.26 ± 0.02	0.35 ± 0.03	1.51 ± 0.37	1.45 ± 0.36
KFE_1_1	0.49	2.22	2.18	0.42	1.23	1.12
KFE_1_2	1.07	2.18	2.07	0.46	1.3	1.25
KFE_1_3	1.1	1.69	1.72	0.46	0.99	1.02
KFE_1_4	0.87	2.21	2.11	0.41	1.21	1.26
KFE_2_1	0.86	2.28	2.16	0.42	1.79	1.54
KFE_2_2	1	2.18	2.02	0.39	1.51	1.48
KFE_2_3	0.98	1.98	1.99	0.38	0.99	1.11
KFE_2_4	1.17	2.06	1.86	0.36	0.88	0.88
Average ± SD	0.94 ± 0.21	2.10 ± 0.19	2.01 ± 0.16	0.41 ± 0.04	1.24 ± 0.30	1.21 ± 0.22
KFF_1_1	0.77	2.33	3.7	0.45	0.65	0.61
KFF_1_2	0.81	2.44	3.74	0.47	0.4	0.37
KFF_1_3	0.84	2.08	3.24	0.35	0.63	0.65
KFF_1_4	0.8	2.12	3.51	0.38	1.1	1.02
KFF_2_1	0.83	2.43	3.64	0.35	0.92	0.91
KFF_2_2	1.01	2.33	3.61	0.43	1.03	0.94
KFF_2_3	1.04	1.99	3.35	0.4	0.97	1.05
KFF_2_4	1.03	2.3	3.27	0.49	1.42	1.37
Average ± SD	0.89 ± 0.11	2.25 ± 0.17	3.51 ± 0.20	0.42 ± 0.05	0.89 ± 0.32	0.87 ± 0.31
Overall average ± SD	0.86 ± 0.10	1.97 ± 0.16	1.89 ± 0.18	0.37 ± 0.05	1.47 ± 0.31	1.45 ± 0.30

Since the amount of starting material for either method was below the optimal amount recommended (1 μg, [7]) for the DNA:adaptors ratio, which ensures that all possible RE fragments would be bound to an adaptor, we proceeded to library construction using these DNA amounts instead of bulking up the random tagged DNA template. However, only ~28 ng of intact gDNA was needed for starting the random tagged amplification by PEP-PCR, which can easily be obtained even from a poor yield DNA preparation, as long as it is of acceptable quality (see Materials and Methods). When no random tagged amplicons are produced by TD-PCR, we have observed that, instead of repeating this step, it is preferable to repeat the PEP-PCR with a new aliquot of the gDNA sample. The reason for this failed tagging step seems independent of the amount or quality of the input gDNA since for two independent experiments performed by two different operators on two plant species we observed 52 failed samples out of 367, and 7 out of 95 (data not shown). We strongly recommend including a water control at the PEP-PCR stage. This sample should yield no product at the TD-PCR step. If positive, we recommend discarding all reagents and starting again.

The overall yield of single end reads per method per lane was 1.97–1.89 million reads by stdGBS and 1.47–1.45 million reads by rtGBS (Table 1). However, a closer inspection of the yield per event revealed some technical errors: all KFD stdGBS libraries in Lane 2 produced an average of 0.26 million reads, but a higher average yield in Lane 1 (1.71 million reads). This unexpectedly low yield indicates a pipetting error, since the samples were simultaneously aliquoted from the library plate into the PCR plate with an 8-tip multichannel pipette. The results shown on Table 1 illustrate how the amount of input gDNA on library preparation has an effect on the number of millions of reads obtained per library; however the yield of mapped BamH I sites to the reference kiwifruit genome ‘Hongyang’ is a more accurate parameter to estimate the value of the total number of reads per library. We chose to report BamH I sites supported by 10 or more reads, since downstream processes for variant calling require well supported data.

**Fig 1:**
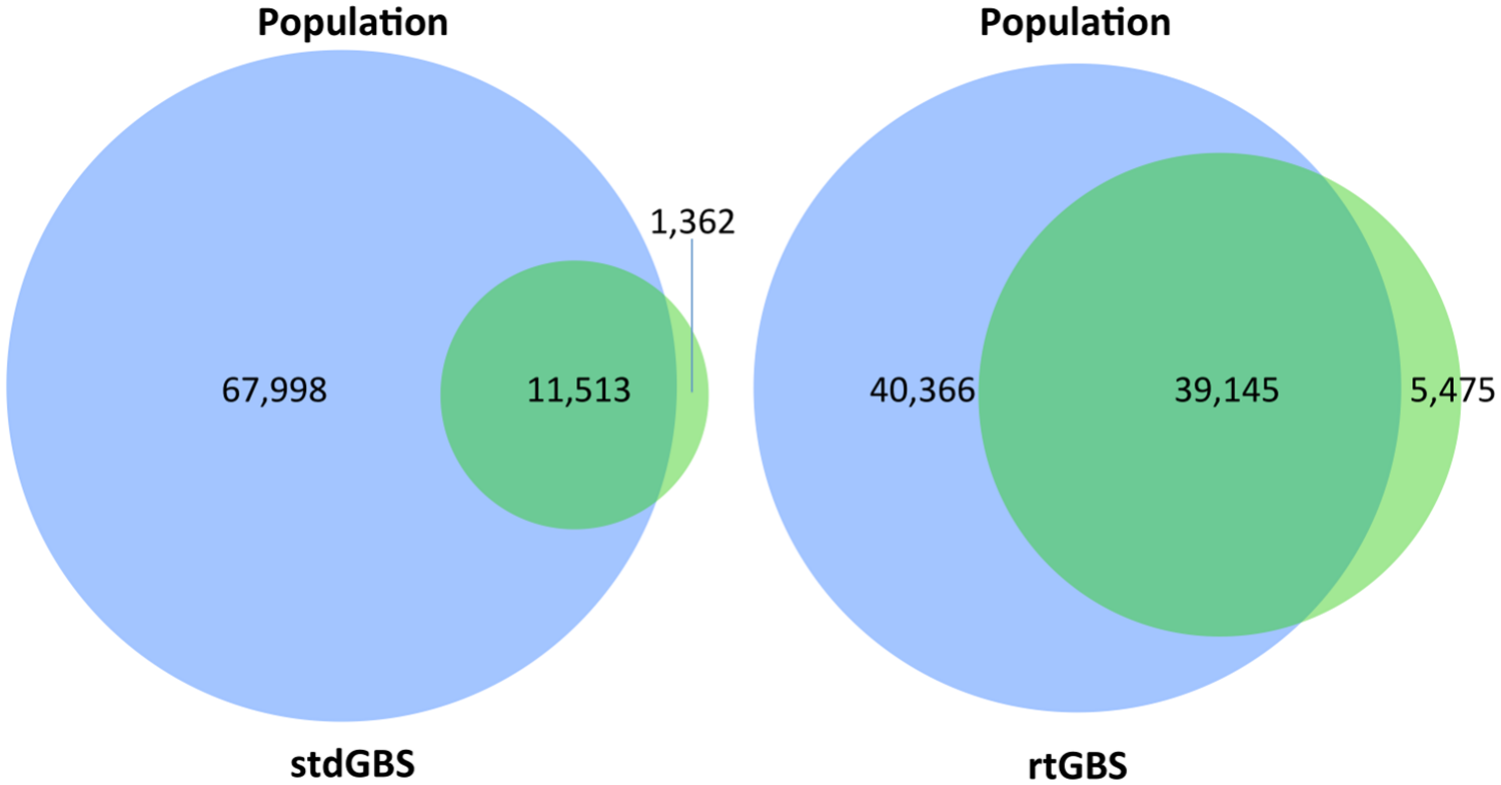
Venn diagrams summarizing the BamH I sites supported by ≥10 reads from the combined datasets of all treatments for stdGBS and rtGBS. The combined datasets were constructed by concatenating all plants_extract_aliquot ([KF.][12][1234]) for both lanes from each library method (green circles). The intersection between the two sets corresponds to the BamH I sites in common by all plants and ‘Hongyang’ denoted as “population” (blue circles).

The kiwifruit ‘Hongyang’ genome draft sequence assembled 616.6 Mbp into 29 pseudochromosomes [22] representing 81.3% of the genome, plus the large pseudochromosome 30, 177 Mbp long, which contains large sections of unknown sequence. The estimated number of BamH I is 79,511, including pseudochromosome 30. The total number of those sites for all experimental treatments found by either stdGBS or rtGBS is summarized in Fig 1. From the predicted 79,511 BamH I sites, rtGBS found 44,620 (39,145 shared with ‘Hongyang’ and 5,475 unique), while stdGBS found 12,875 (11,513 shared with ‘Hongyang’ and 1,362 unique). This corresponds to 3.4 more sites found by rtGBS than by stdGBS. The additional BamH I sites found by each method can be explained by the inherent differences between these six genotypes and ‘Hongyang’.

**Fig 2:**
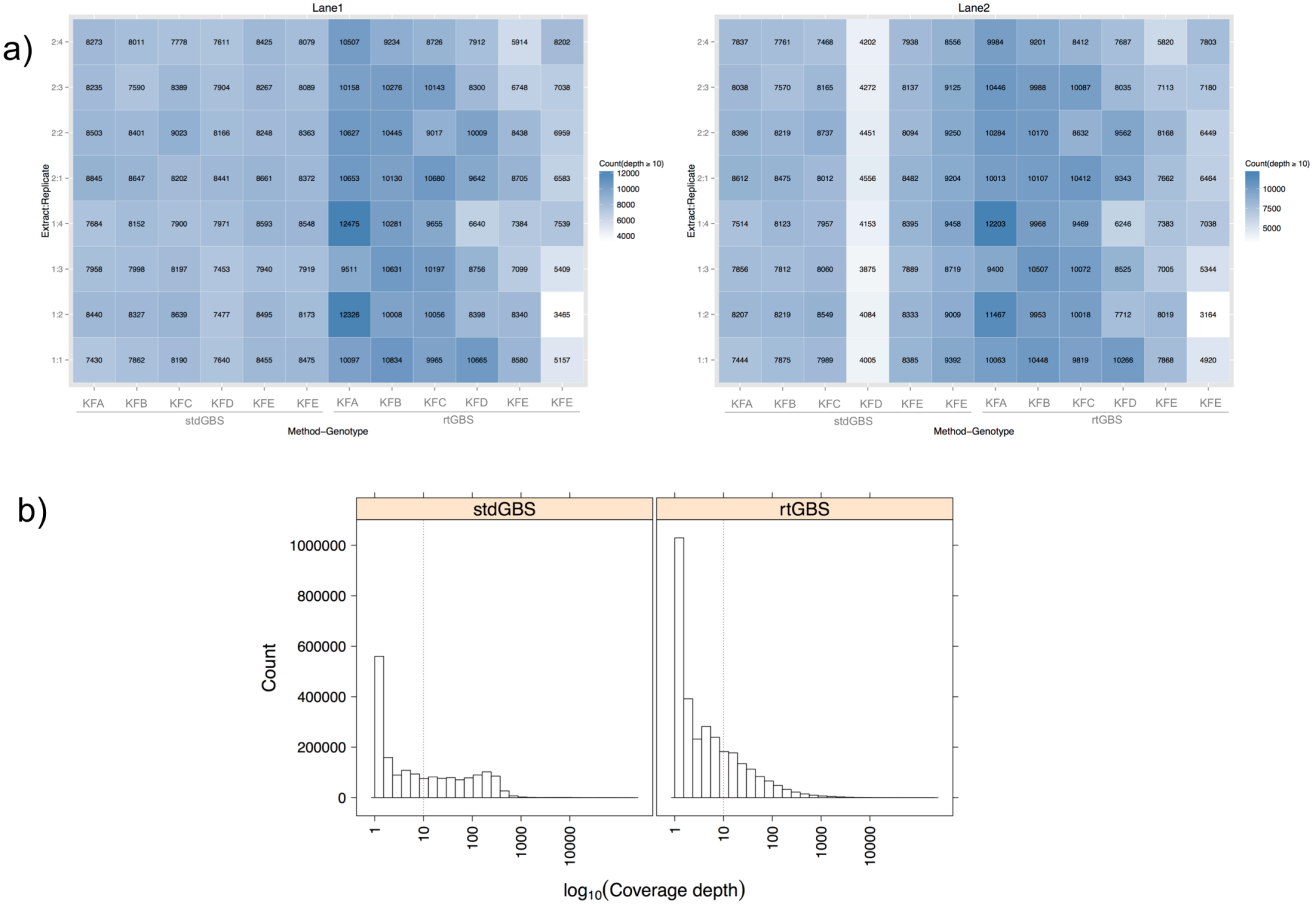
Difference on coverage depth obtained by stdGBS and rtGBS. A) Heatmaps of the mapped BamH I sites covered by ≥10 reads. B) Read coverage depth of the concatenated dataset for each library method. The cut-off value of 10 reads is marked with a red dotted line.

The results examined by treatment are summarized on the heatmaps shown in Fig 2a. Even though more BamH I sites were found by rtGBS compared to stdGBS, different trends are observed among the different plant_extraction_sample cases. For plants KFA, KFB, KFC and KFD (except KFC_2_2, both lanes, and KFD_1_4, Lane 1), all rtGBS libraries mapped more reads. Sample KFD_1_4 by either method on lane 2 cannot be assessed since all stdGBS libraries were subsampled at the library amplification stage, as explained above. The situation is different for KFE and KFF. In this case, only the following rtGBS libraries found more BamH I sites: KFE_1_1 (Lane 1) and KFE_2_1 (Lane 2), KFE_2_2 (both lanes), and KFF_2_4 (Lane 1). One explanation for this result is that for all KFE and KFF stdGBS libraries we obtained an average of 2.01–3.51 million reads, which is above the overall average for this method. However the number of mapped BamH I sites supported by 10 reads or more is more than twice by rtGBS than for stdGBS, as summarized for all plant_extraction_sample cases in Fig 2b.

### BamH I fragment distribution mapped to the kiwifruit ‘Hongyang’ genome

**Fig 3:**
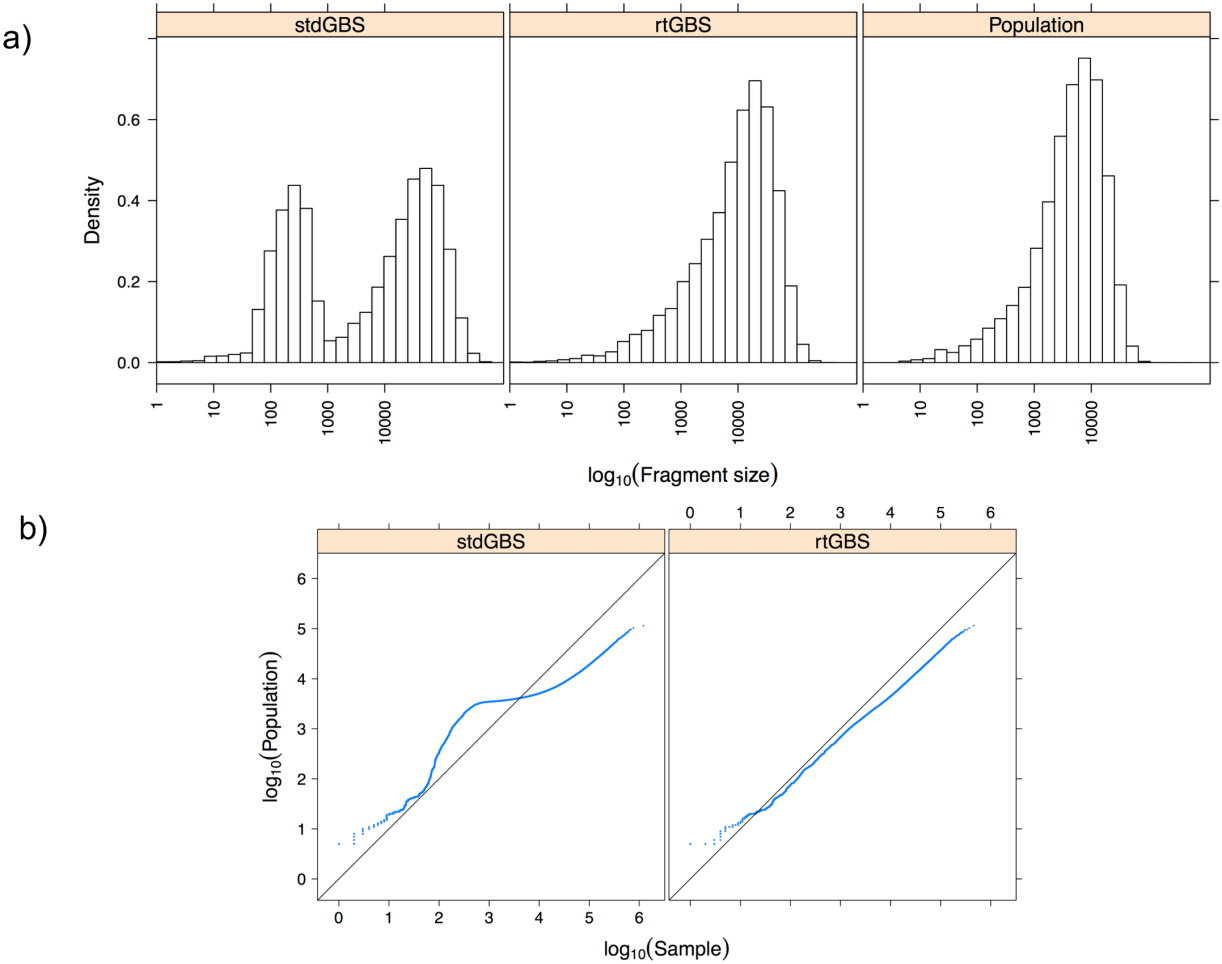
Fragment size distribution analysis. A) Semi-logarithmic display of the BamH I fragment size distribution among the ‘Hongyang’ genome (population) and the two library methods. B) Probability distribution plots for the two GBS library methods vs. Hongyang genome RE fragment size distribution.

The distribution of RE sites in the genome determines the chances of mapping valuable traits in the population. The distribution of the BamH I RE fragment in ‘Hongyang’ (denoted as “Population” in Fig 3a) behaved in an exponential fashion and so appears relatively unbiased, as did the RE fragments targeted by rtGBS. In contrast, the RE fragments generated by stdGBS libraries were distributed in a bimodal way, i.e. demonstrating a strong bias for a subset of all possible BamH I sites.

To compare the RE fragment probability distributions between ‘Hongyang’ genome vs. each GBS library method (sample), we constructed their corresponding quantile plots (Fig 3b). The points on the quantile plot for rtGBS method lie very close to the line y = x, i.e., their distribution is very similar to the one observed on the ‘Hongyang’ genome, while the quantile plot for stdGBS showed a strong fragment size bias for this sampling method.

**Fig 4:**
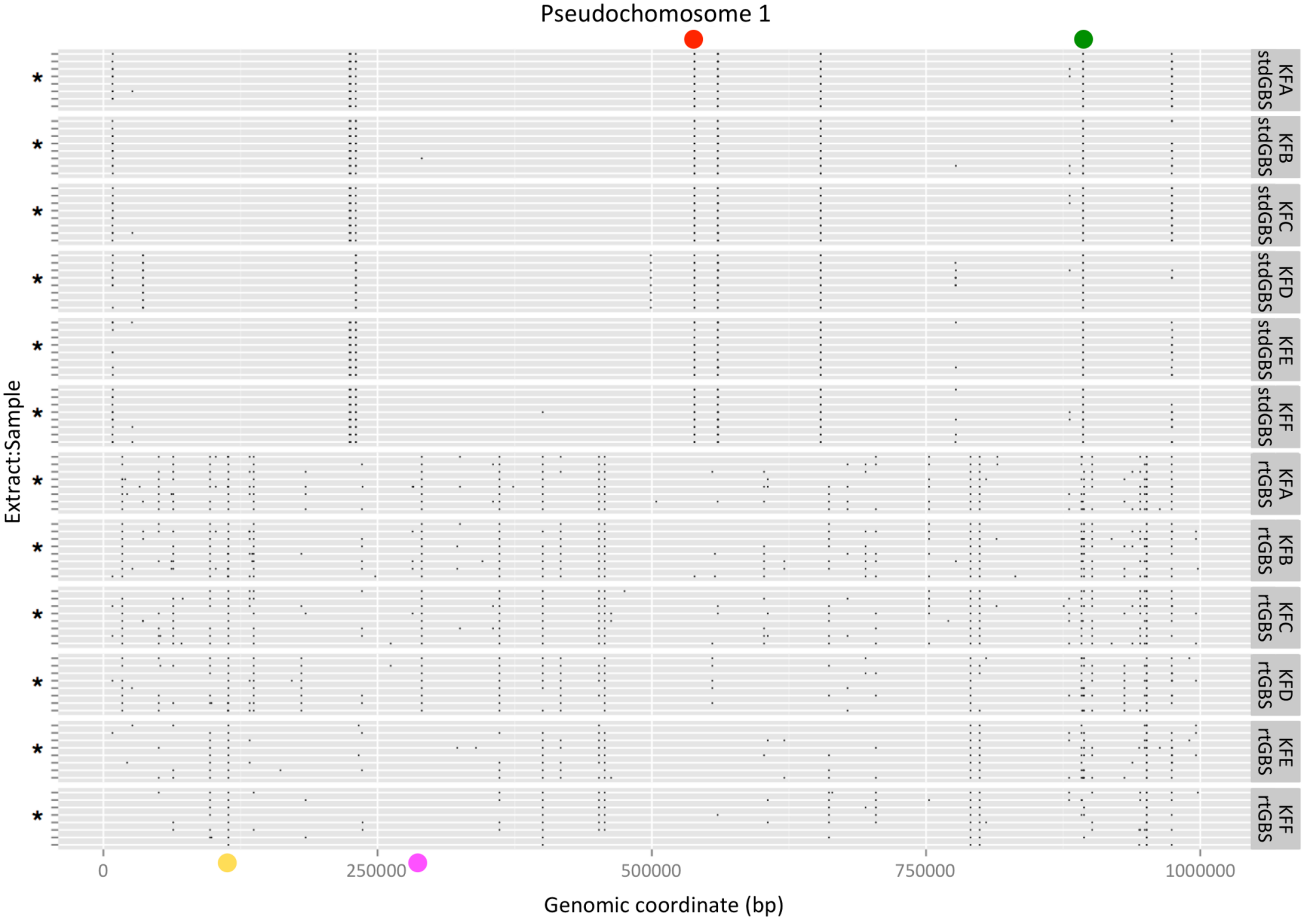
Physical representation of the first 1 Mbp of pseudochromosome 1 in ‘Hongyang’ genome sequence with mapped reads found by stdGBS and rtGBS methods, all treatments, supported by ≥10 reads. The occurrence of BamH I sites per method and/or plant is denoted by coloured dots: Sites found in all treatments by stdGBS and rtGBS (green dot), found by either one type of library (red and yellow dots), and sites poorly represented by KFE and KFF (pink dot). The extract_aliquot data is denoted by an asterisk, in the following order from top to bottom of each plant_method panel: 2_4, 2_3, 2_2, 2_1, 1_4, 1_3, 1_2, 1_1.

A visual display of the mapped BamH I sites supported by ≥10 reads for all treatments on the first 1 Mbp of pseudochromosome 1 is shown in Fig 4. Overall, more BamH I sites were mapped by rtGBS. Most of the few BamH I sites mapped by stdGBS are represented in all treatments, while rtGBS mapped more sites in a scattered fashion. The small proportion of BamH I sites found by stdGBS occupied a larger “real estate” than the portion found by rtGBS from all available reads. The average total number of reads for stdGBS libraries per lane was 92.6 million (1.93 million reads x 48 libraries, Table 1) and 70.1 million for rtGBS (1.46 million reads x 48 libraries), which corresponded to 48% and 37% of usable reads, respectively. However, those ratios were partitioned disproportionately among the mapped BamH I reads between the libraries since by stdGBS only 14% of all possible BamH I sites were found (11,513 Fig 1), whereas by rtGBS 49% of BamH I sites were found in a smaller sequence set (39,145 (Fig 1), 70.1 million reads). The rtGBS libraries from KFF performed poorly, as noted before in Fig 2 heatmaps. A similar landscape is observed for the first 1 Mbp of all the other pseudochromosomes (S1 Fig).

### Variability analysis of BamH I RE site counts in relation to biological and technical replicates

Table 2 provides estimates of fixed effect means and variance components of random effects obtained by fitting two alternative models, the generalized linear mixed model (GLMM), and the linear mixed model (LMM), to the observed number of BamH I sites collected on 48 experiment units x 2 methods x 2 lanes.

**Table 2:** Best linear unbiased estimates of fixed effects (method:lane) and estimated random effect (plant:sample (extract:aliquot)) variances were obtained by fitting the Generalized Linear Mixed Model (GLMM) and Linear Mixed Model (LMM) and their standard error.

			Estimate (Ln)	
Effect			GLMM	LMM
*Fixed*				
	Method	rtGBS	10.37 ± 0.052	10.36 ± 0.055
		stdGBS	9.83 ± 0.052	9.82 ± 0.055
	Lane	1	10.12 ± 0.038	10.11 ± 0.040
		2	10.08 ± 0.038	10.07 ± 0.040
*Random*				
	Plant		0.0	0.0
	Sample:Extract (Plant)		0.0008 ± 0.0008	0.0009 ± 0.0009
	Plant:Method		0.0157 ± 0.0074	0.0172 ± 0.0080
	Residual		0.0106 ± 0.0013	0.0112 ± 0.0014

All estimates and their standard errors were on the natural logarithm scale. The largest discrepancy was a plant:method interaction due to variable method performance. The random effect sample:extract described the variation in technical replicates which was an order of magnitude smaller. The rtGBS libraries had significantly higher counts than stdGBS, although there was some variability in between plant samples. We compared the two cumulative distribution functions for stdGBS and rtGBS of the number of BamH I sites observed for both methods by performing a two-sample Kolmogorov-Smirnov test, which was significantly well supported (*p*-value < 2.2x10^-16^). The BamH I sites sampled by the two GBS library methods showed a very different fragment distribution compared to the population (‘Hongyang’ genome). More details of the modeling, the parameter estimates (presented on natural log scale) between the two models discussed in Table 2 were very similar, and consistent within reported margins of error, are included in S1 Text.

## Discussion

We have characterized significant differences between two methods for preparing GBS libraries: GBS with intact gDNA based on Elshire et al. [1] and by random tagging. Overall, more high quality sequencing reads were obtained by stdGBS than by rtGBS, but the rtGBS method mapped more BamH I sites against the kiwifruit draft genome sequence than stdGBS with an RE fragment size distribution more evenly, closely resembling the expected BamH I fragment population observed in the genome of the ‘Hongyang’ variety of kiwifruit (Fig 3). Concerns about possible bias introduced by the large number of cycles needed for random tagging DNA at the PEP-PCR stage can be discarded since the unbiased RE fragment size distribution (Fig 3) indicated this is not a methodological issue.

The number of sites with sufficient read depth usable for genetic analysis is an important factor since a sufficient read depth is required to distinguish accurately between homozygous and heterozygous single nucleotide polymorphisms (SNPs). A sufficient number of reads are required to determine with confidence whether any site is truly homozygous as opposed to appearing homozygous due to shallow sampling of only one of the two alleles. Where rare cutting RE is used, more of the RE sites will be present on larger fragments and are more likely to be missed by stdGBS than rtGBS.

The ability to sample RE sites evenly by rtGBS across the whole genome is an important modification to the stdGBS method since it allows a wider choice of RE including rare cutters (recognition sites of eight or more bases). It will also enable better planning of GBS experiments, by incorporating information on the genome size, the number of cut sites (either theoretical using the RAD_counter tool [8] or predicted using Biostrings [26]), the number of samples to be run in a single lane, and the actual read depth expected after processing the raw reads. Careful planning will lead to a more accurate SNP calling results. In this study we settled for a moderately frequent RE cutter, BamH I, since the kiwifruit genome contains 75,726 BamH I sites (estimated based on the haploid genome size of 758 Mbp obtained by flow cytometry [28] and the GC content, using RAD_counter) that allowed pooling 96 samples with a minimum of 12 reads supporting each RE site. Other enzymes with identical number of RE sites are: Age I, Mlu I, Nco I, Avr II, BsiW I, Pvu I, Pst I, Nhe I, Acc65 I, Sal I, ApaL I, Aat II, Sac I, Sph I, Bmt I, Kpn I and BspE I. We chose BamH I for its readily availability and reliable performance. Other popular restriction enzymes often found in a basic molecular biology laboratory, such as Hind III or EcoR I, cut too frequently on the kiwifruit genome (258,869 RE sites) and do not fit the basic criteria of minimum number of reads per site under the multiplex conditions selected. The aim of random tagging GBS is to target RE sites that could not be efficiently captured by stdGBS, i.e., the restriction fragments produced are larger than the optimal size required for cluster formation during parallel sequencing. Enzymes such as EcoR I and Hind III fall into this category, and theoretically would only have 4 reads supporting each RE in the kiwifruit genome if 96 samples are pooled for sequencing.

Another important difference between the two methods is the cost associated with preparing the libraries which for large studies involving several hundred samples can become a limiting step. DNA extractions can consume a substantial portion of the overall budget in any GBS experiment, as well as time. The quality and quantity of gDNA is crucial for the success in preparing a standard GBS library. Often the best quality gDNA is obtained with commercial kits, which simplify and standardize the extraction process but can also add to the overall cost of the experiment. Standard GBS libraries require ~ 1 μg gDNA, which is within the average yield for many plant mini preparations. Excluding standard laboratory consumables and labour, we estimated the cost of one standard GBS library to be USD 7.9. The initial cost of one rtGBS library is USD 2 more expensive than stdGBS. In this experiment, one gDNA preparation could produce ~ 89 PEP-PCR reactions enough to prepare ~4,450 TD-PCR reactions (2,500 ng/28 ng per PEP-PCR) (50 TD-PCR per PEP-PCR) and ~ 2,225 rtGBS libraries (see average yields stated in Material and Methods). Once we produce a set of random tagged amplicons containing the GBS common adaptor on both ends for a gDNA preparation, these amplicons can be used to produce GBS libraries with any restriction enzyme we select for a particular study. In comparison, stdGBS would require a new gDNA extraction to produce GBS libraries with a different restriction enzyme.

## Conclusion

The random tagging GBS method allowed for better sampling and representation of restriction sites than standard GBS. The additional steps required by rtGBS are technically simple and the two methods have comparable initial costs. The final significant advantage of rtGBS is the reduction of intact gDNA required and the ability of producing a considerably larger amount of template for library preparation with the GBS common adaptor on both ends. This allows the construction of GBS libraries with any RE overhang required for future experiments at reduced cost. Overall, the advantages contributed by the rtGBS technique are a significant improvement over stdGBS.

The random tagging GBS method should also enable better application of GBS to polyploid plants, where there will be more RE sites than in the corresponding diploid and a much greater read depth from the use of rare cutters will be required to accurately call SNPs and minimize the number of sequencing lanes required. When faced with large genomes and polyploidy issues, the use of frequent enzyme cutter is not advisable. Also, attention needs to be given to which portion of the genome will be targeted: gene-rich single copy sites, heterochromatic areas, untranslated regions, etc., since different traits are regulated by different parts of the genome. Random tagging GBS allows the researcher to retrieve rare restriction sites, easily amplifiable by PCR and within the optimal construct size for efficient cluster formation during parallel sequencing.

## Supporting Information

S1 Fig Hongyang Pseudochromosomes 2 to 30.pdf. Physical representation of the first 1 Mbp of pseudochromosome 2 to 30 in ‘Hongyang’ draft genome sequence with mapped reads found by stdGBS and rtGBS methods, all treatments, supported by ≥10 reads.
(PDF)

S1 Text Analysis of rt GBS Expt2 NdS notes.pdf. Further details of the modeling, the parameter estimates (presented on natural log scale) between the two models discussed in Table 2.
(PDF)
