# Supplementary material for: Random Tagging Genotyping by Sequencing (rtGBS), an Unbiased Approach to Locate Restriction Enzyme Sites across the Target Genome
Source: PLoS One. 2015 Dec 3;10(12):e0143193. doi: 10.1371/journal.pone.0143193 (PMC4669186; doi:10.1371/journal.pone.0143193)
Supplement: S1 Fig — Physical representation of the first 1 Mbp of pseudochromosome 2 to 30 in ‘Hongyang’ draft genome sequence with mapped reads found by stdGBS and rtGBS methods, all treatments, supported by ≥10 reads. (PDF) [file pone.0143193.s001.pdf]

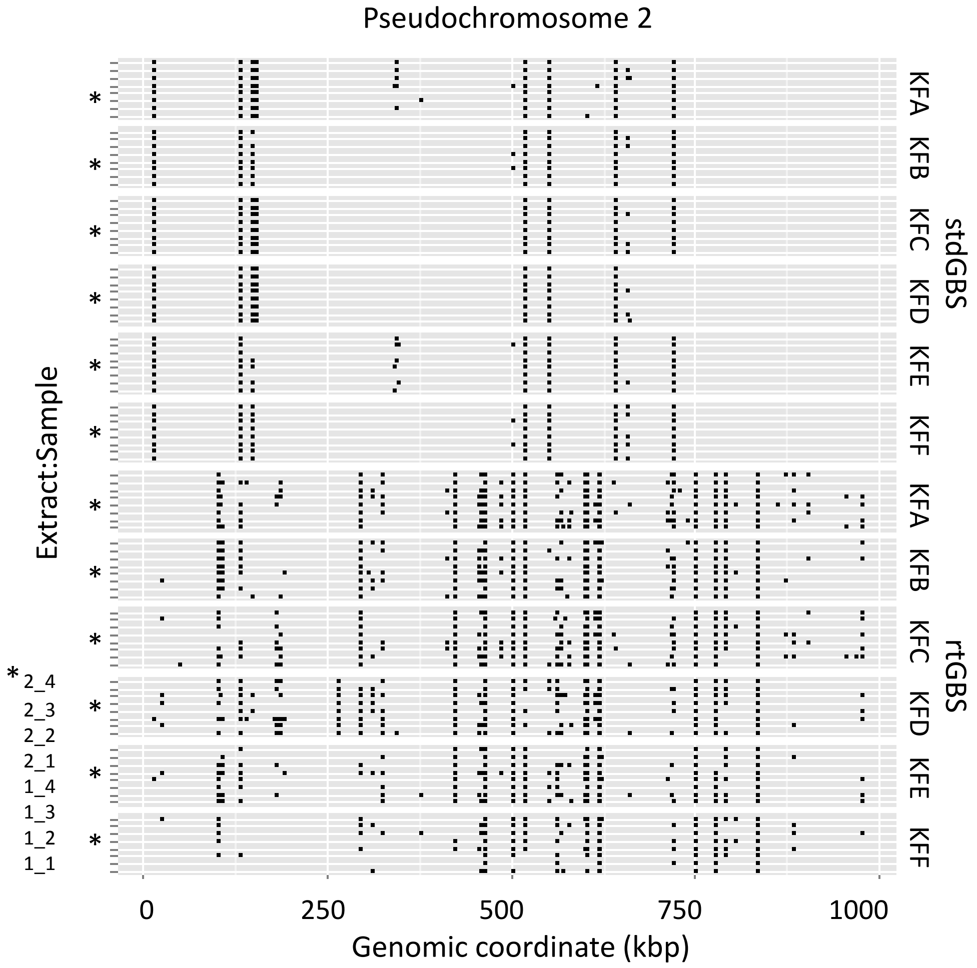

Pseudochromosome 2
Extract:Sample
KFA
KFB
KFC
KFD
KFE
KFF
stdGBS
KFA
KFB
KFC
KFD
KFE
KFF
rtGBS
2_4
2_3
2_2
2_1
1_4
1_3
1_2
1_1
0
250
500
750
1000
Genomic coordinate (kbp)

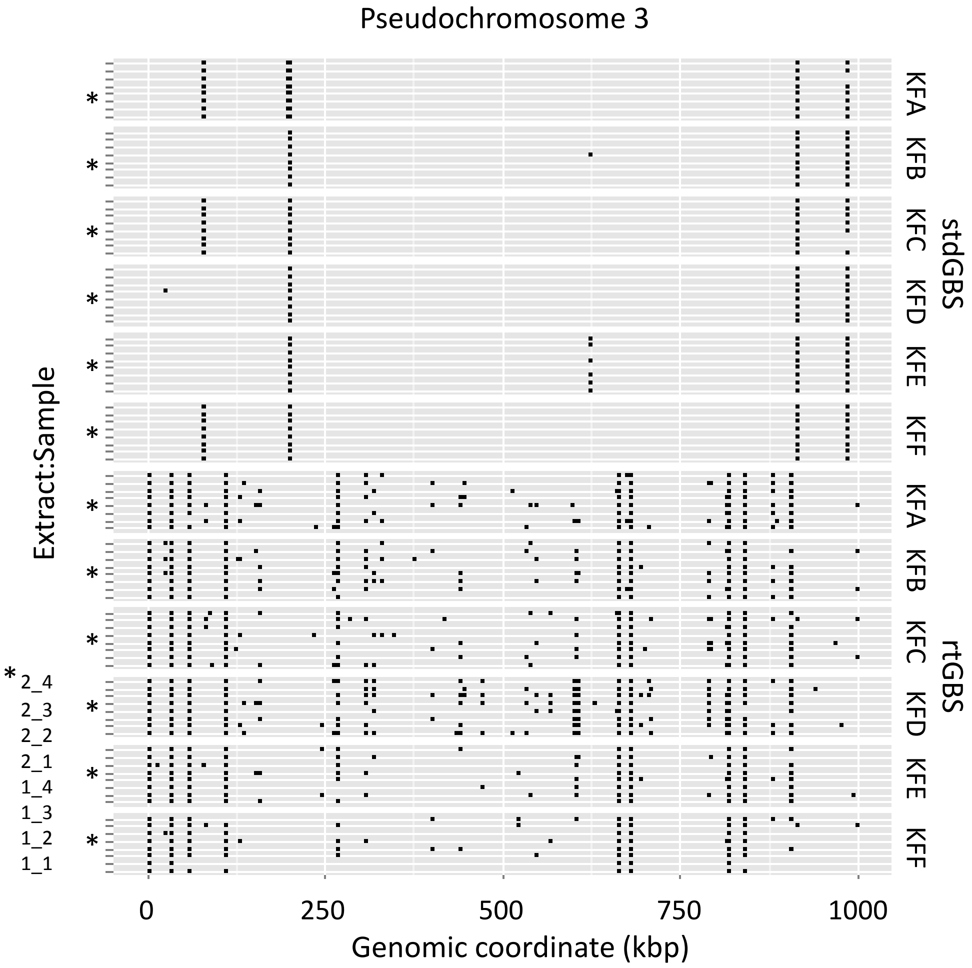

Pseudochromosome 3
Extract:Sample
stdGBS
rtGBS
KFA
KFB
KFC
KFD
KFE
KFF
2_4
2_3
2_2
2_1
1_4
1_3
1_2
1_1
0
250
500
750
1000
Genomic coordinate (kbp)

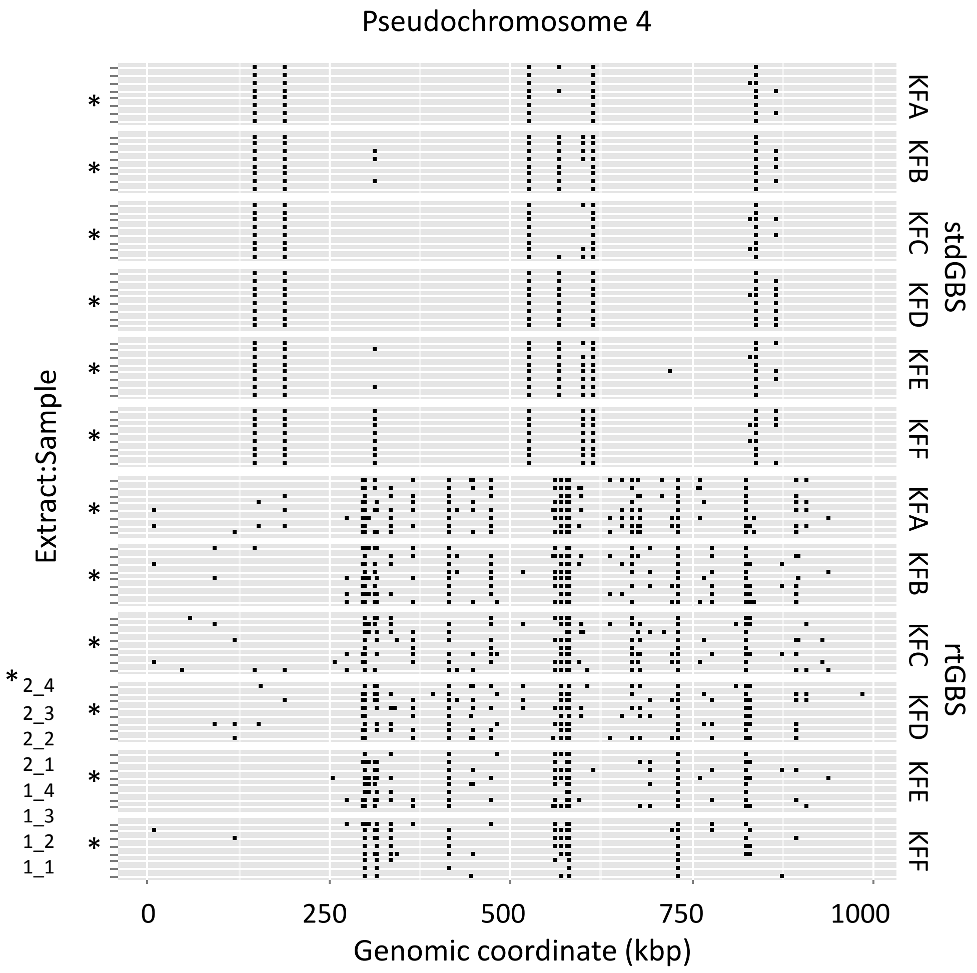

Pseudochromosome 4
KFA
KFB
KFC
KFD
KFE
KFF
stdGBS
KFA
KFB
KFC
KFD
KFE
KFF
rtGBS
Extract:Sample
2_4
2_3
2_2
2_1
1_4
1_3
1_2
1_1
0
250
500
750
1000
Genomic coordinate (kbp)

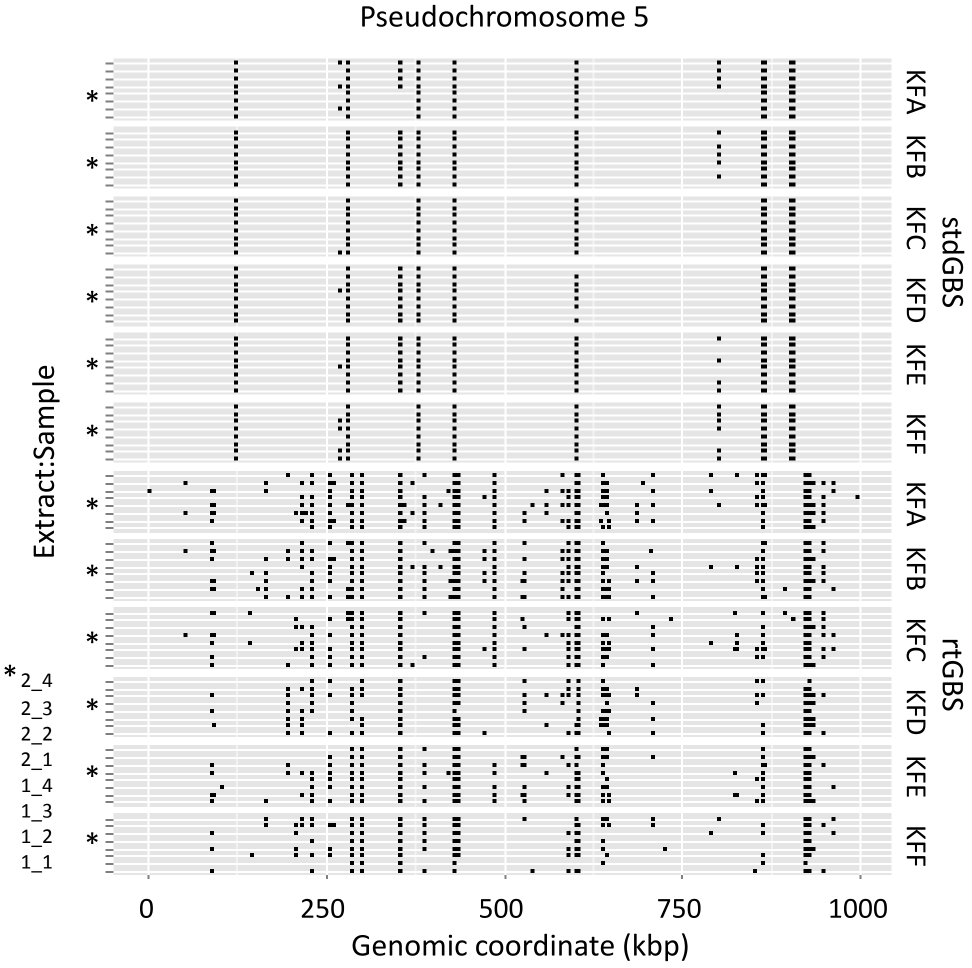
Pseudochromosome 5
Extract:Sample
stdGBS
rtGBS
KFA
KFB
KFC
KFD
KFE
KFF
2_4
2_3
2_2
2_1
1_4
1_3
1_2
1_1
0
250
500
750
1000
Genomic coordinate (kbp)

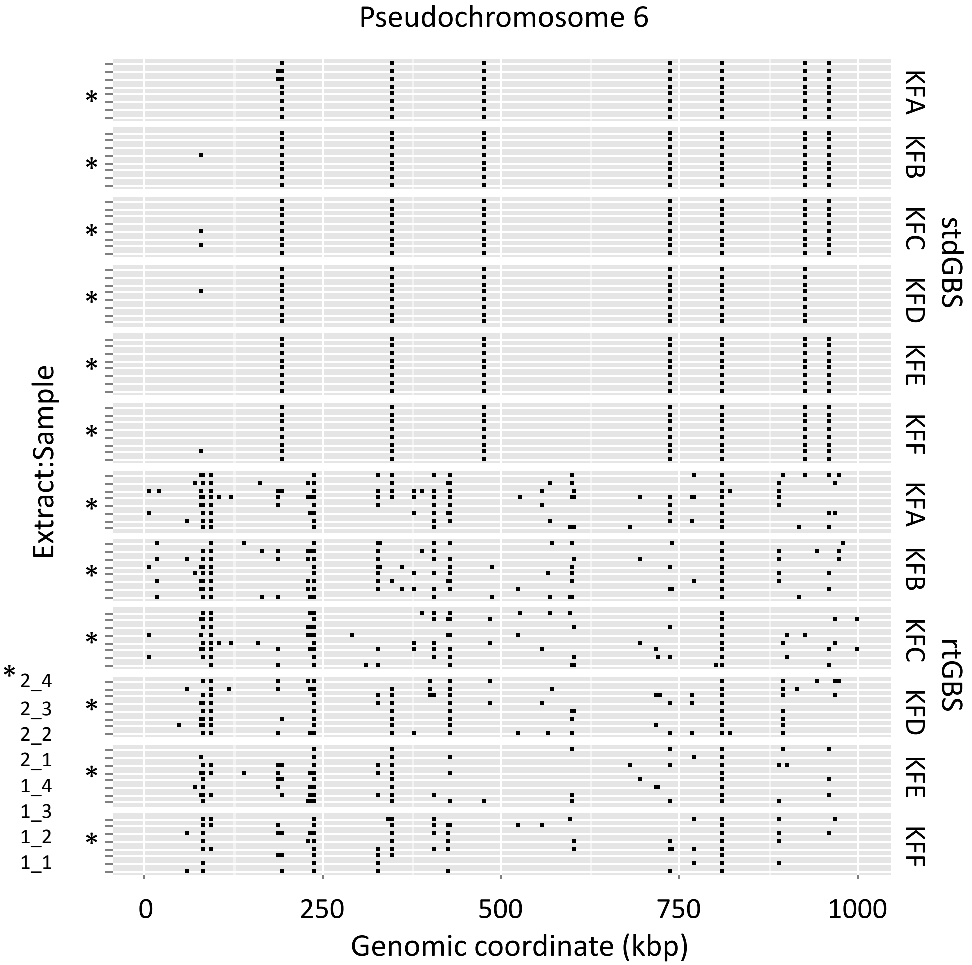
Pseudochromosome 6
Extract:Sample
stdGBS
rtGBS
KFA
KFB
KFC
KFD
KFE
KFF
*
2_4
2_3
2_2
2_1
1_4
1_3
1_2
1_1
0
250
500
750
1000
Genomic coordinate (kbp)

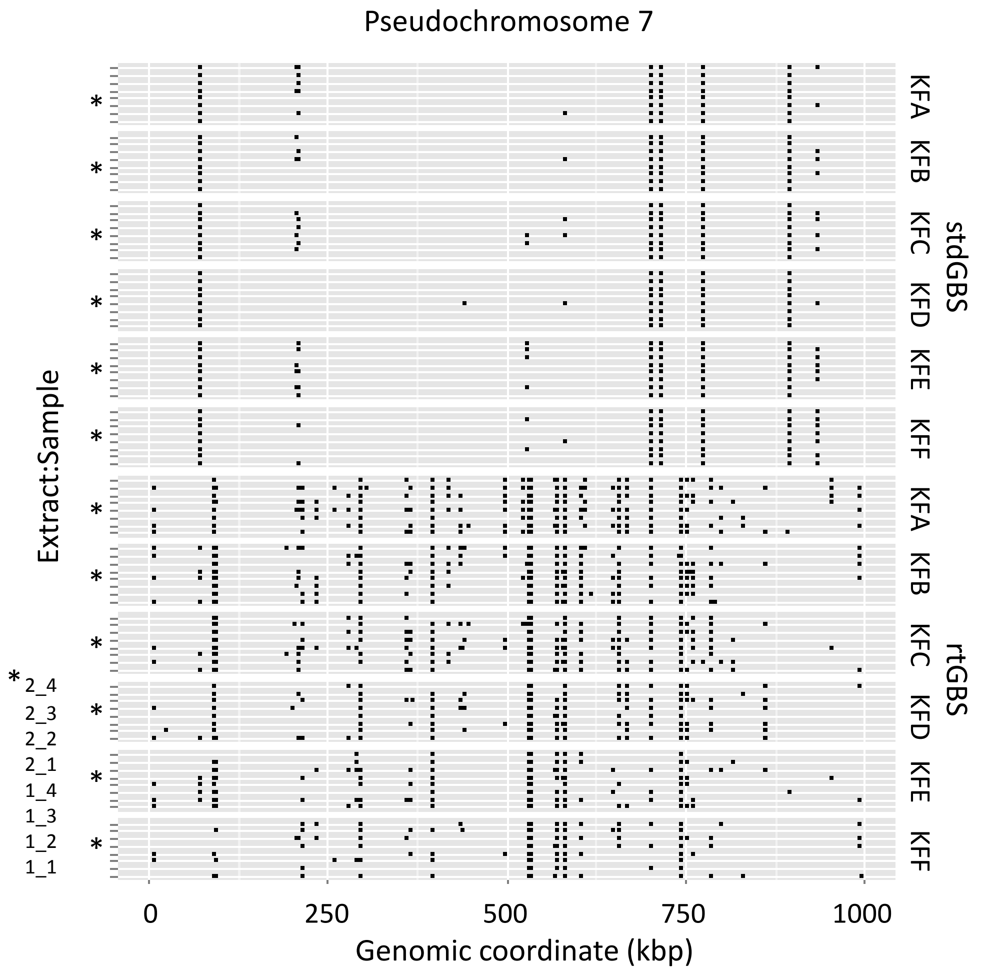
Pseudochromosome 7
Extract:Sample
stdGBS
rtGBS
KFA
KFB
KFC
KFD
KFE
KFF
KFA
KFB
KFC
KFD
KFE
KFF
*2_4
2_3
2_2
2_1
1_4
1_3
1_2
1_1
0
250
500
750
1000
Genomic coordinate (kbp)

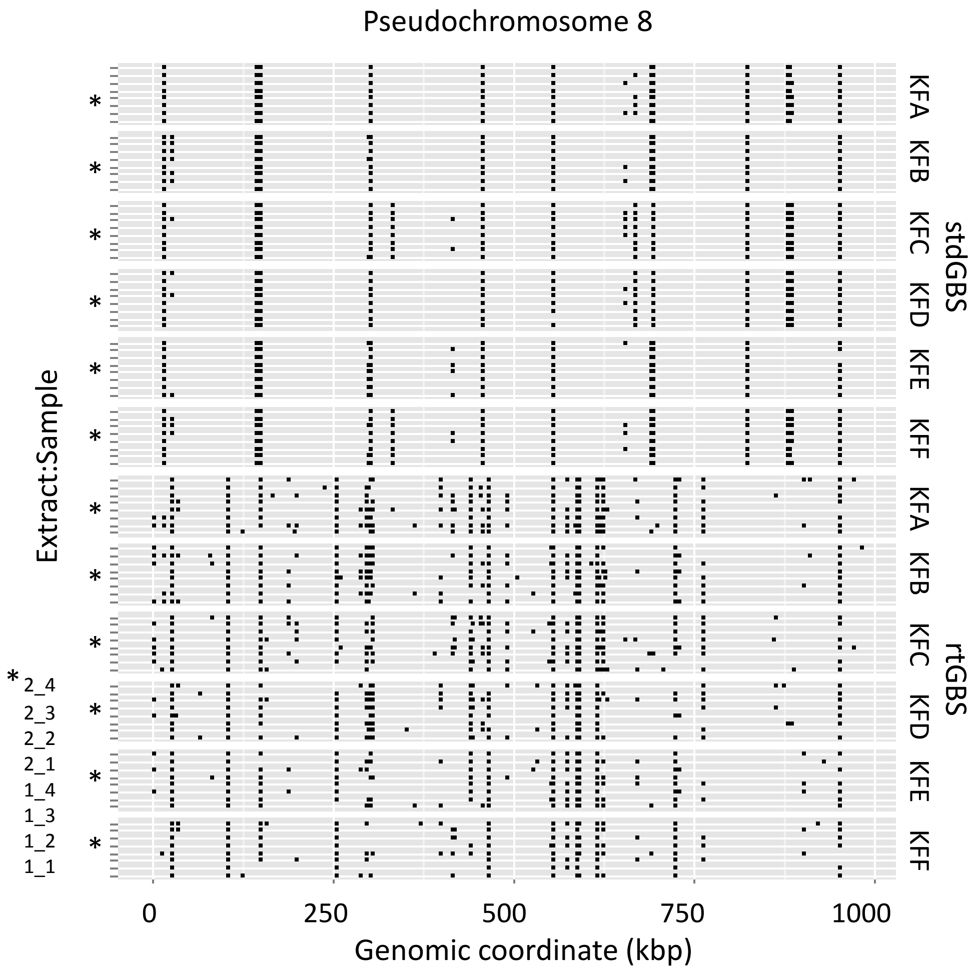

Pseudochromosome 8
Extract:Sample
*
*2_4
2_3
2_2
2_1
1_4
1_3
1_2
1_1
KFA
KFB
KFC
KFD
KFE
KFF
stdGBS
rtGBS
0
250
500
750
1000
Genomic coordinate (kbp)

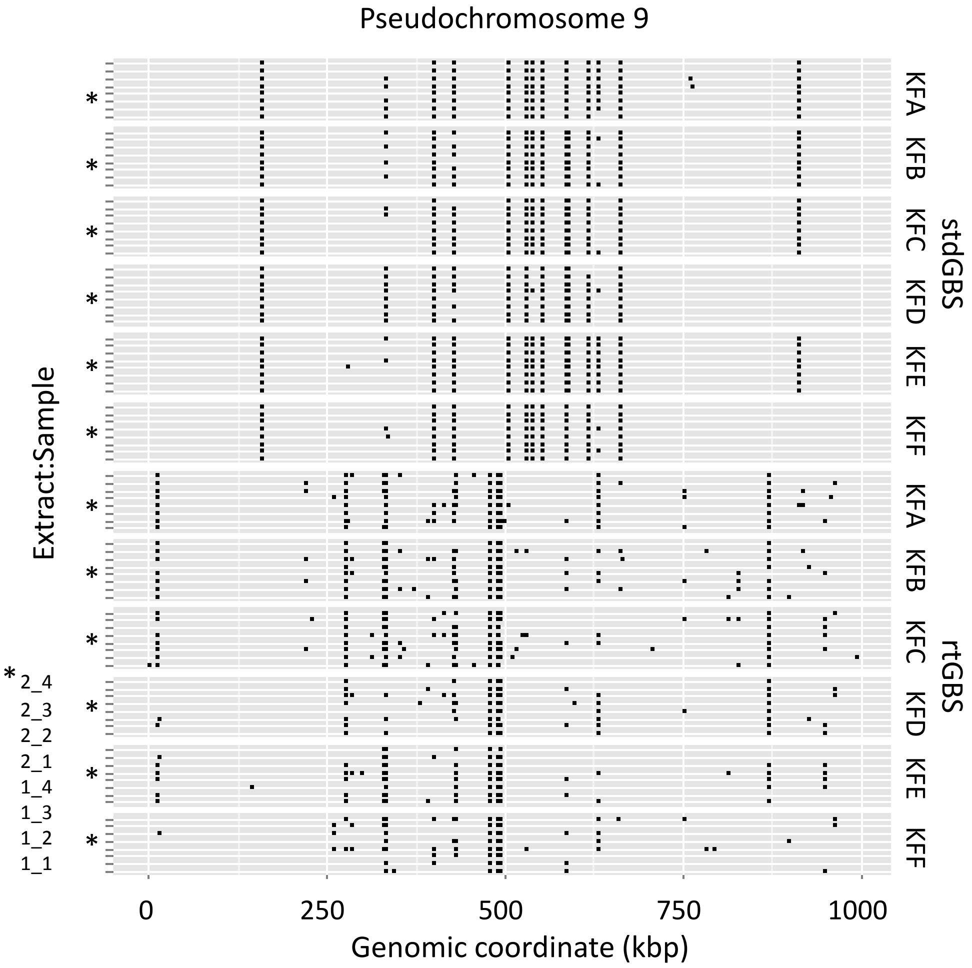

Pseudochromosome 9
Extract:Sample
KFA
KFB
KFC
KFD
KFE
KFF
stdGBS
KFA
KFB
KFC
KFD
KFE
KFF
rtGBS
*2_4
2_3
2_2
2_1
1_4
1_3
1_2
1_1
0
250
500
750
1000
Genomic coordinate (kbp)

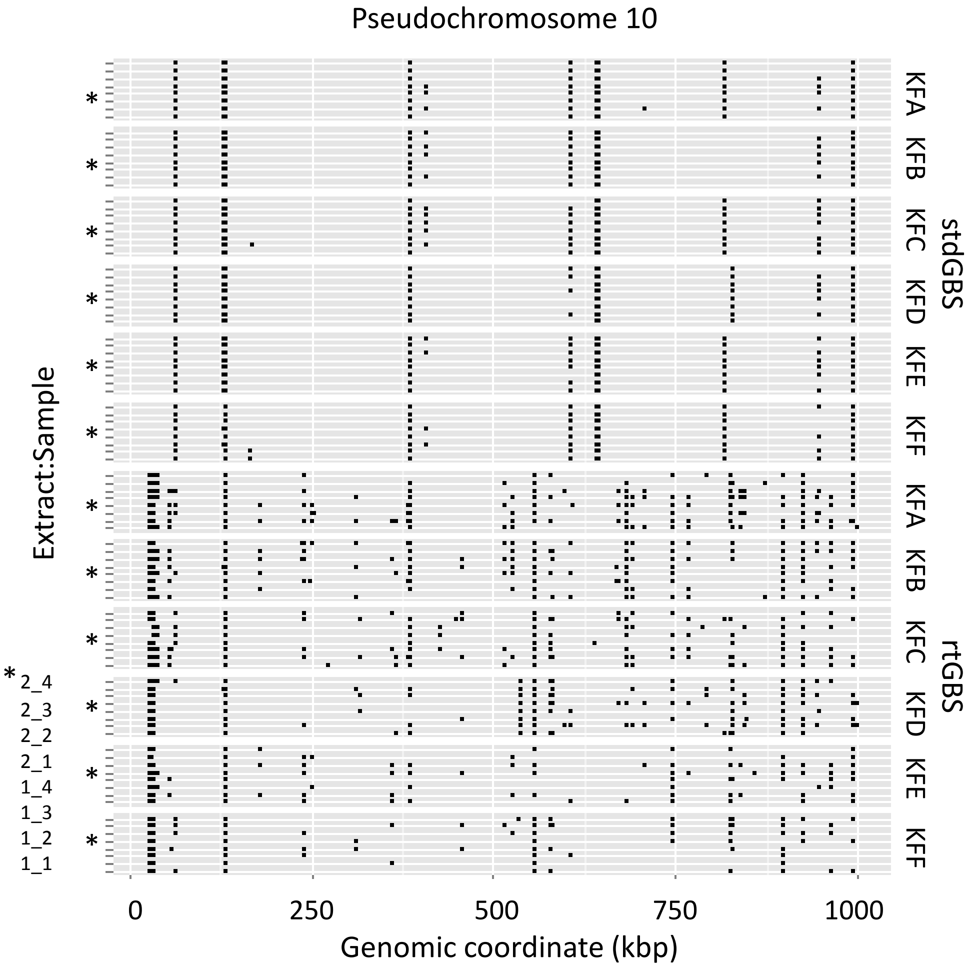

Pseudochromosome 10
Extract:Sample
stdGBS
rtGBS
KFA
KFB
KFC
KFD
KFE
KFF
2_4
2_3
2_2
2_1
1_4
1_3
1_2
1_1
0
250
500
750
1000
Genomic coordinate (kbp)

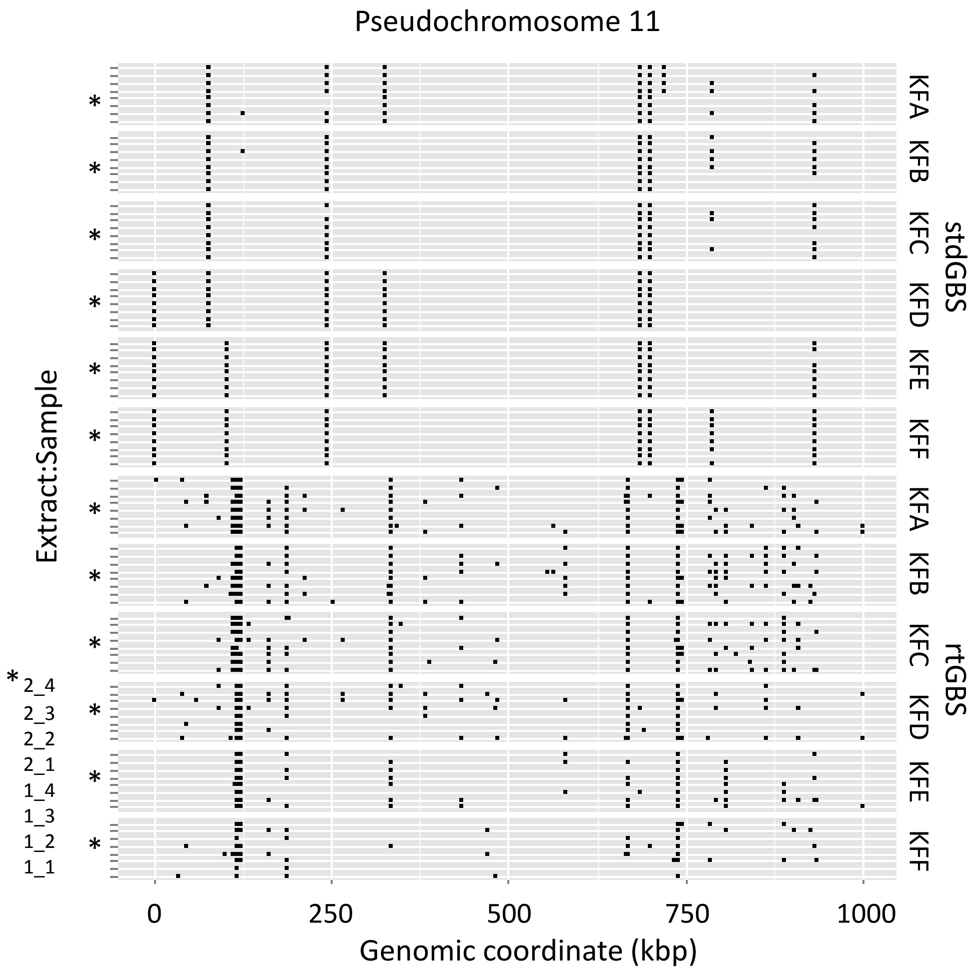

Pseudochromosome 11
Extract:Sample
stdGBS
rtGBS
KFA
KFB
KFC
KFD
KFE
KFF
KFA
KFB
KFC
KFD
KFE
KFF
*2_4
2_3
2_2
2_1
1_4
1_3
1_2
1_1
0
250
500
750
1000
Genomic coordinate (kbp)

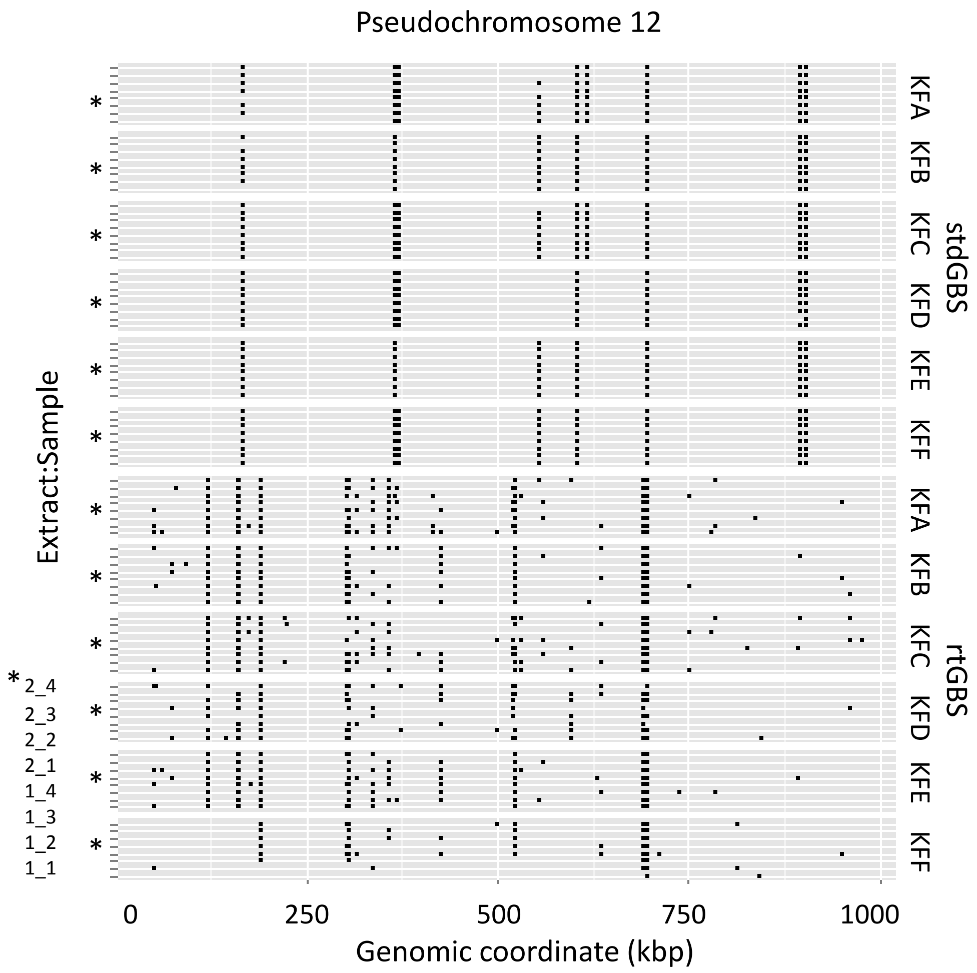

Pseudochromosome 12
Extract:Sample
2_4
2_3
2_2
2_1
1_4
1_3
1_2
1_1
KFA
KFB
KFC
KFD
KFE
KFF
stdGBS
rtGBS
0
250
500
750
1000
Genomic coordinate (kbp)

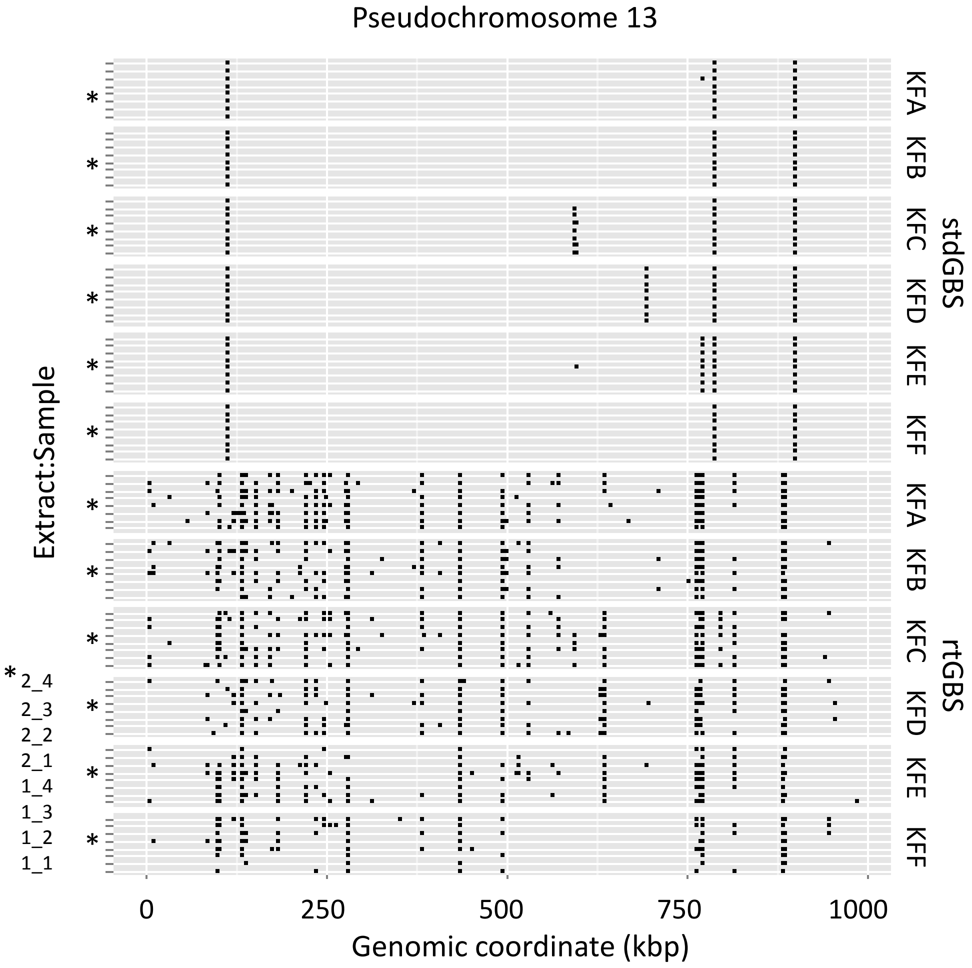
Pseudochromosome 13
Extract:Sample
2_4
2_3
2_2
2_1
1_4
1_3
1_2
1_1
KFA
KFB
KFC
KFD
KFE
KFF
stdGBS
rtGBS
0
250
500
750
1000
Genomic coordinate (kbp)

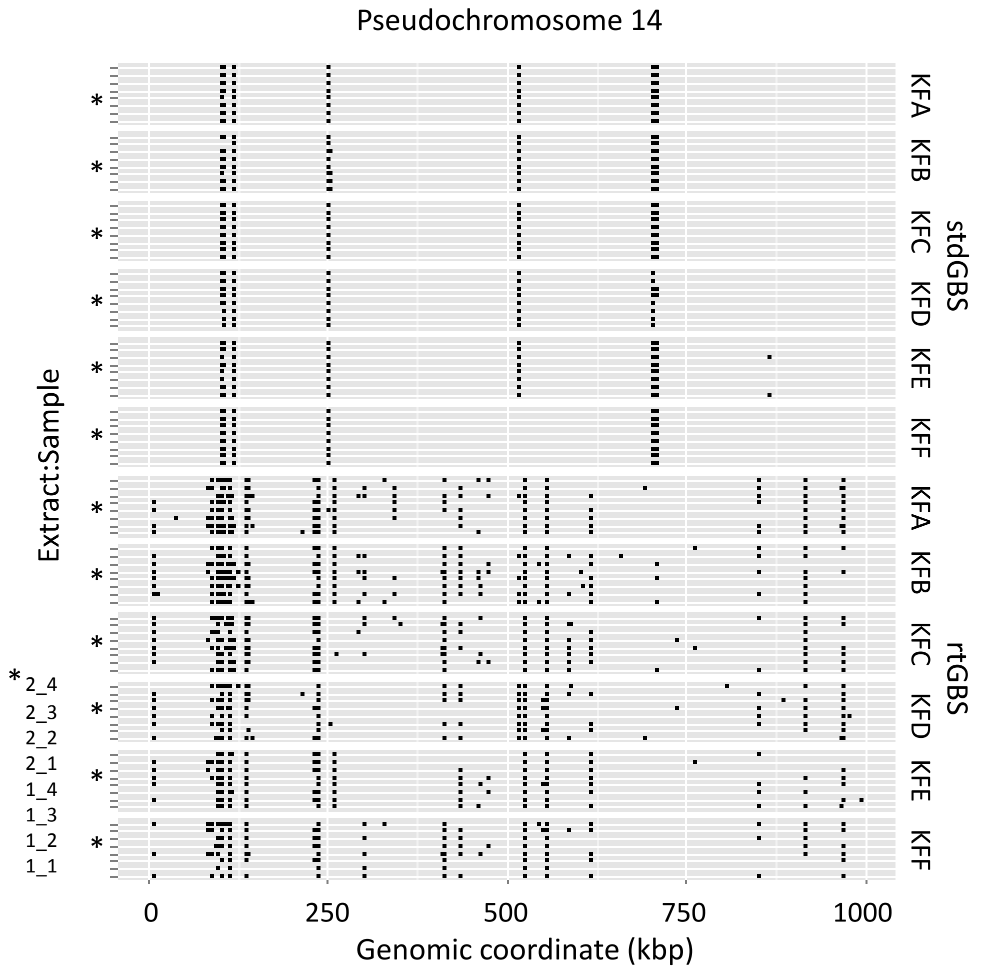

Pseudochromosome 14
Extract:Sample
stdGBS
rtGBS
KFA
KFB
KFC
KFD
KFE
KFF
2_4
2_3
2_2
2_1
1_4
1_3
1_2
1_1
0
250
500
750
1000
Genomic coordinate (kbp)

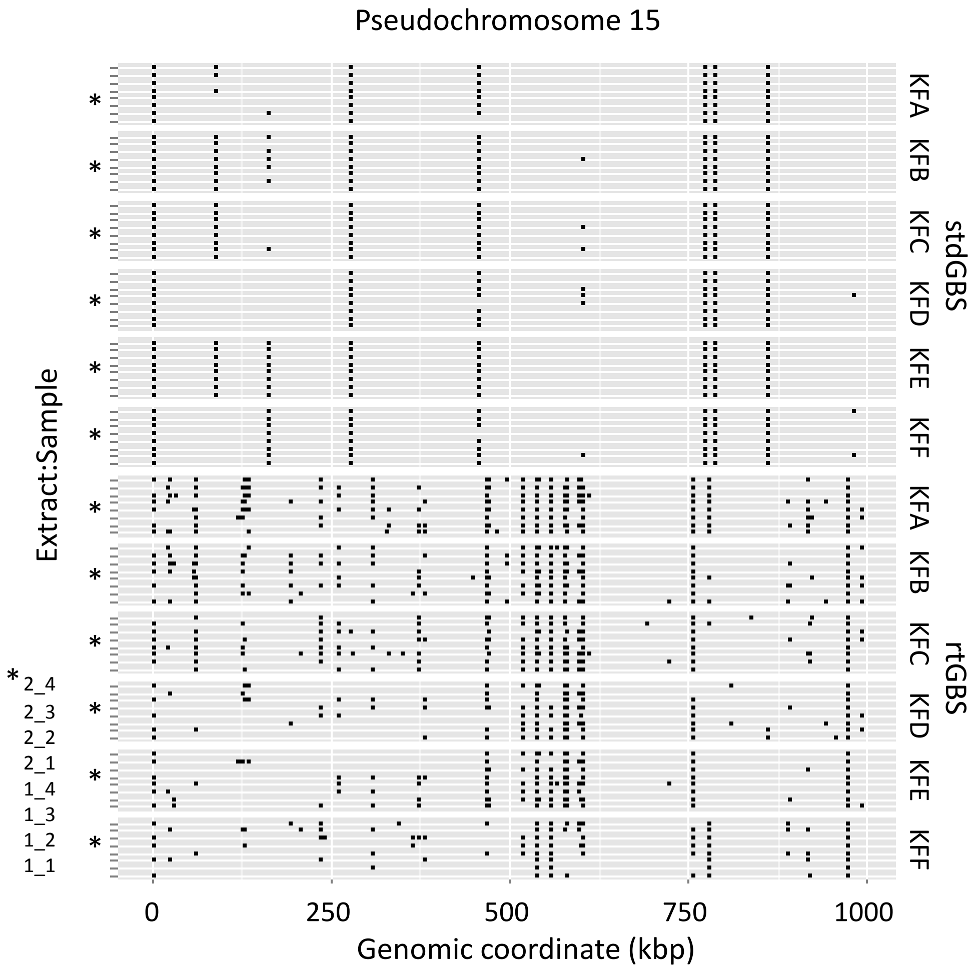

Pseudochromosome 15
Extract:Sample
KFA
KFB
KFC
KFD
KFE
KFF
stdGBS
rtGBS
*2_4
2_3
2_2
2_1
1_4
1_3
1_2
1_1
0
250
500
750
1000
Genomic coordinate (kbp)

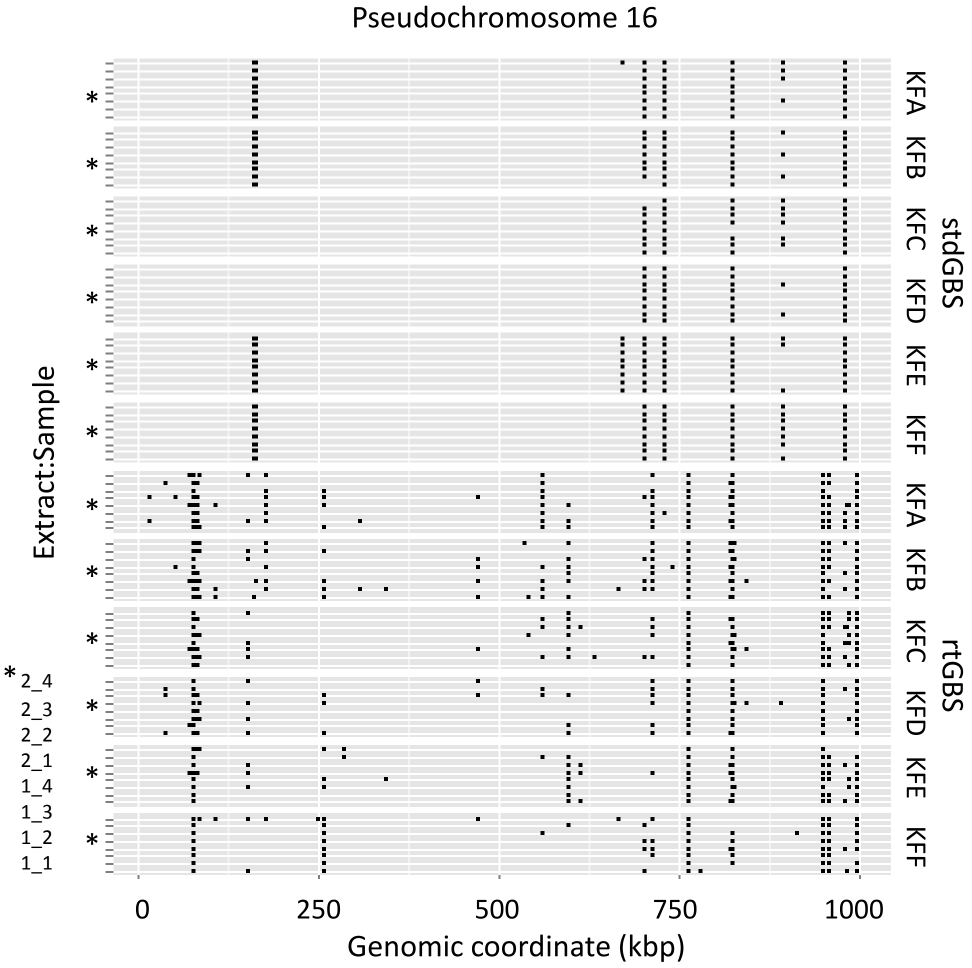

Pseudochromosome 16
Extract:Sample
KFA
KFB
KFC
KFD
KFE
KFF
stdGBS
rtGBS
2_4
2_3
2_2
2_1
1_4
1_3
1_2
1_1
0
250
500
750
1000
Genomic coordinate (kbp)

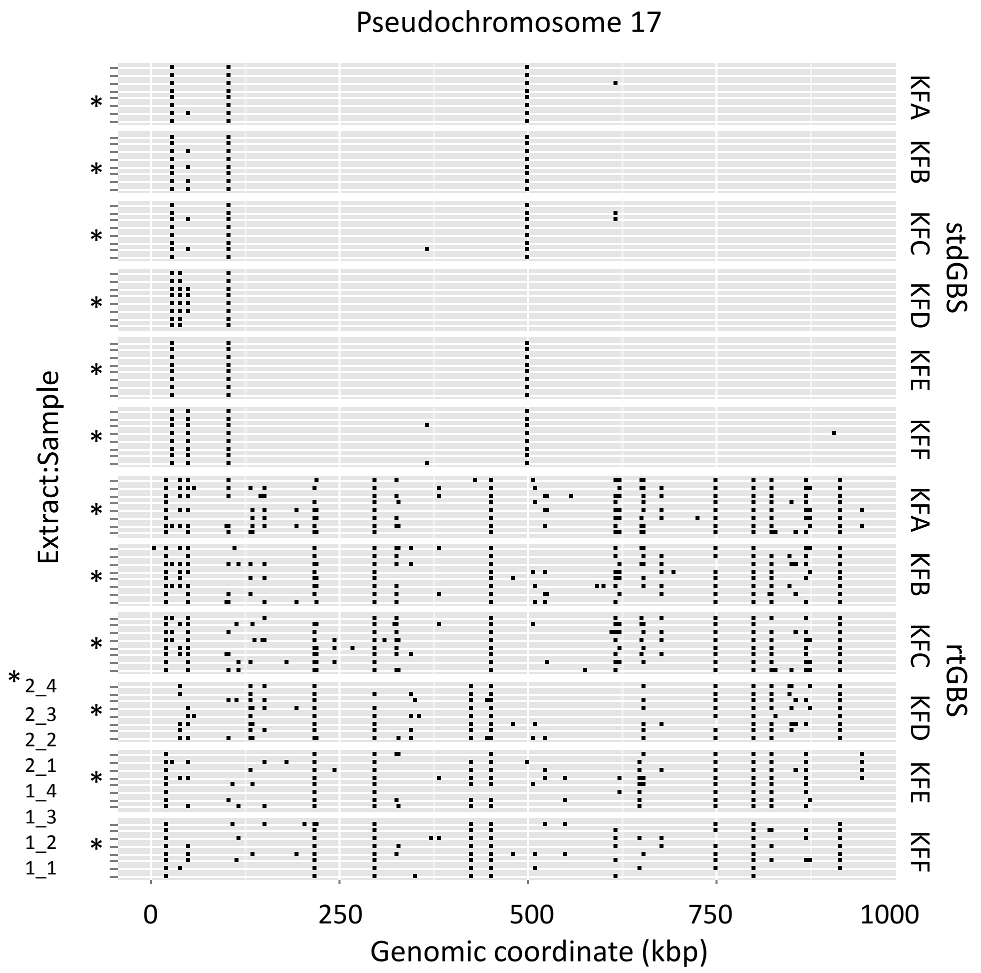

Pseudochromosome 17
Extract:Sample
stdGBS
rtGBS
KFA
KFB
KFC
KFD
KFE
KFF
KFA
KFB
KFC
KFD
KFE
KFF
2_4
2_3
2_2
2_1
1_4
1_3
1_2
1_1
0
250
500
750
1000
Genomic coordinate (kbp)

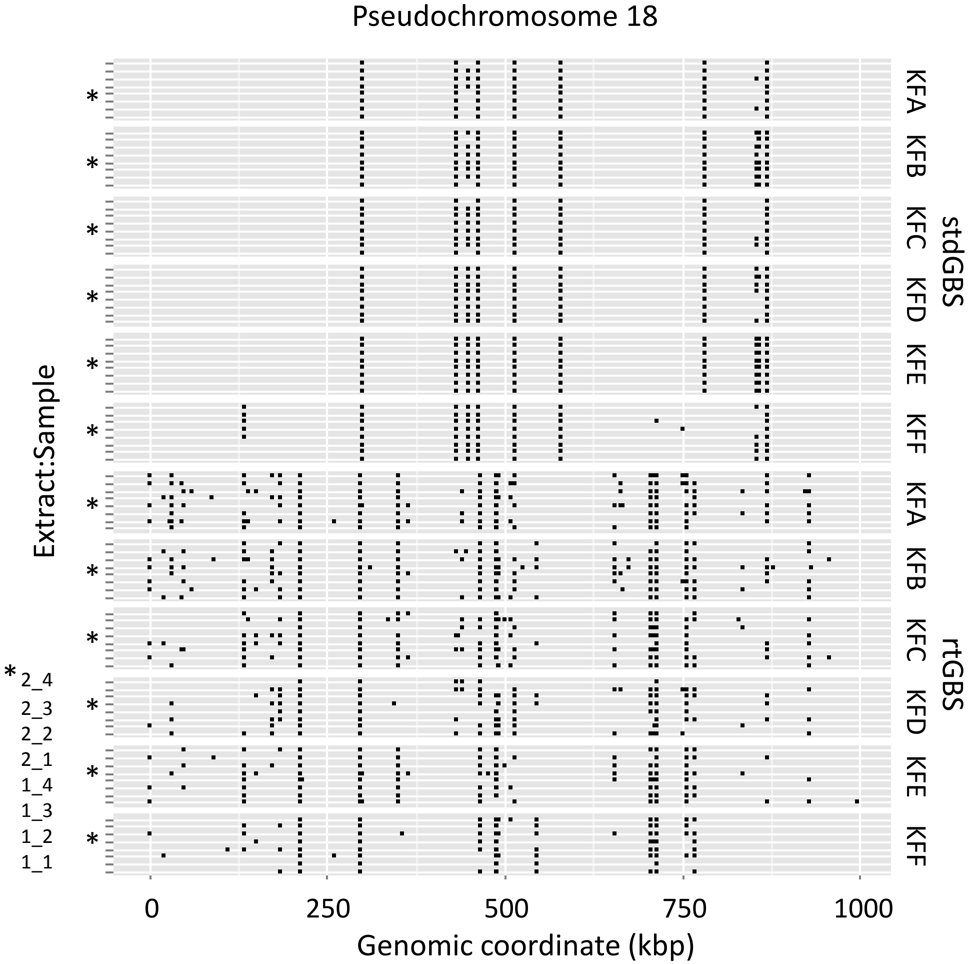

Pseudochromosome 18
Extract:Sample
stdGBS
rtGBS
KFA
KFB
KFC
KFD
KFE
KFF
2_4
2_3
2_2
2_1
1_4
1_3
1_2
1_1
0
250
500
750
1000
Genomic coordinate (kbp)

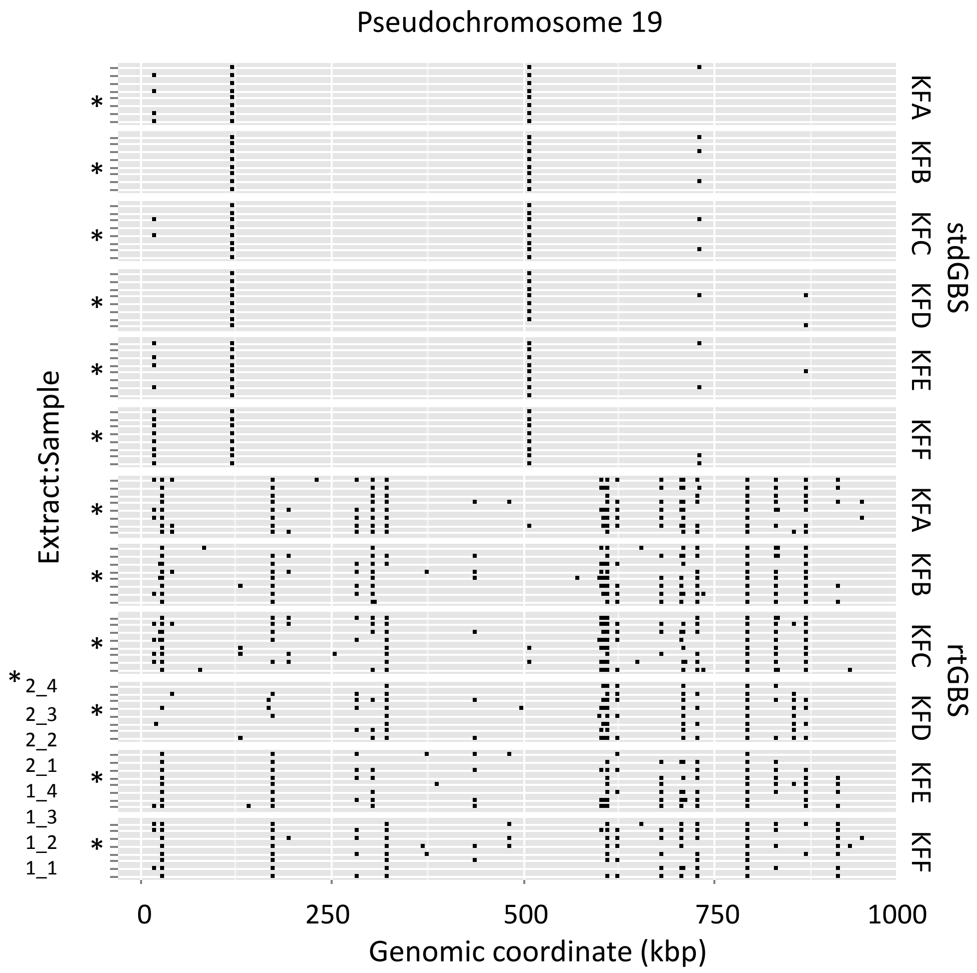

Pseudochromosome 19
Extract:Sample
stdGBS
rtGBS
KFA
KFB
KFC
KFD
KFE
KFF
KFA
KFB
KFC
KFD
KFE
KFF
2_4
2_3
2_2
2_1
1_4
1_3
1_2
1_1
0
250
500
750
1000
Genomic coordinate (kbp)

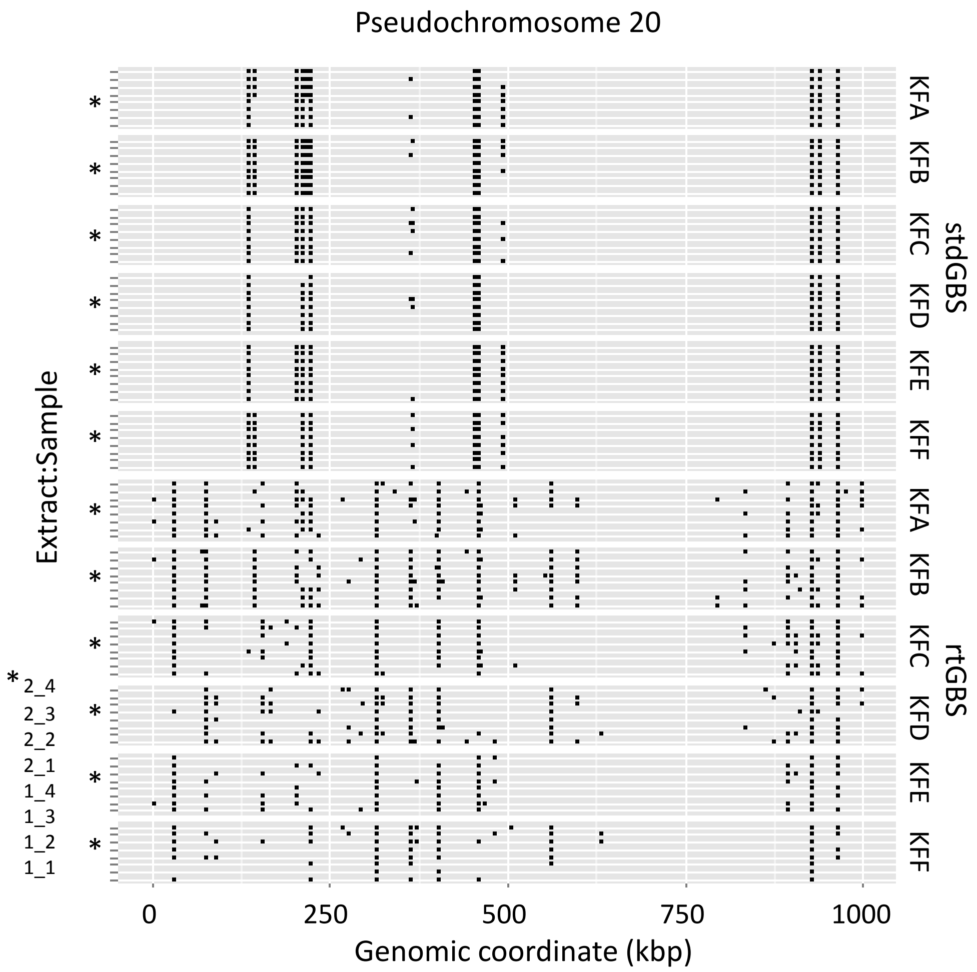

Pseudochromosome 20
Extract:Sample
KFA
KFB
KFC
KFD
KFE
KFF
stdGBS
rtGBS
*
2_4
2_3
2_2
2_1
1_4
1_3
1_2
1_1
0
250
500
750
1000
Genomic coordinate (kbp)

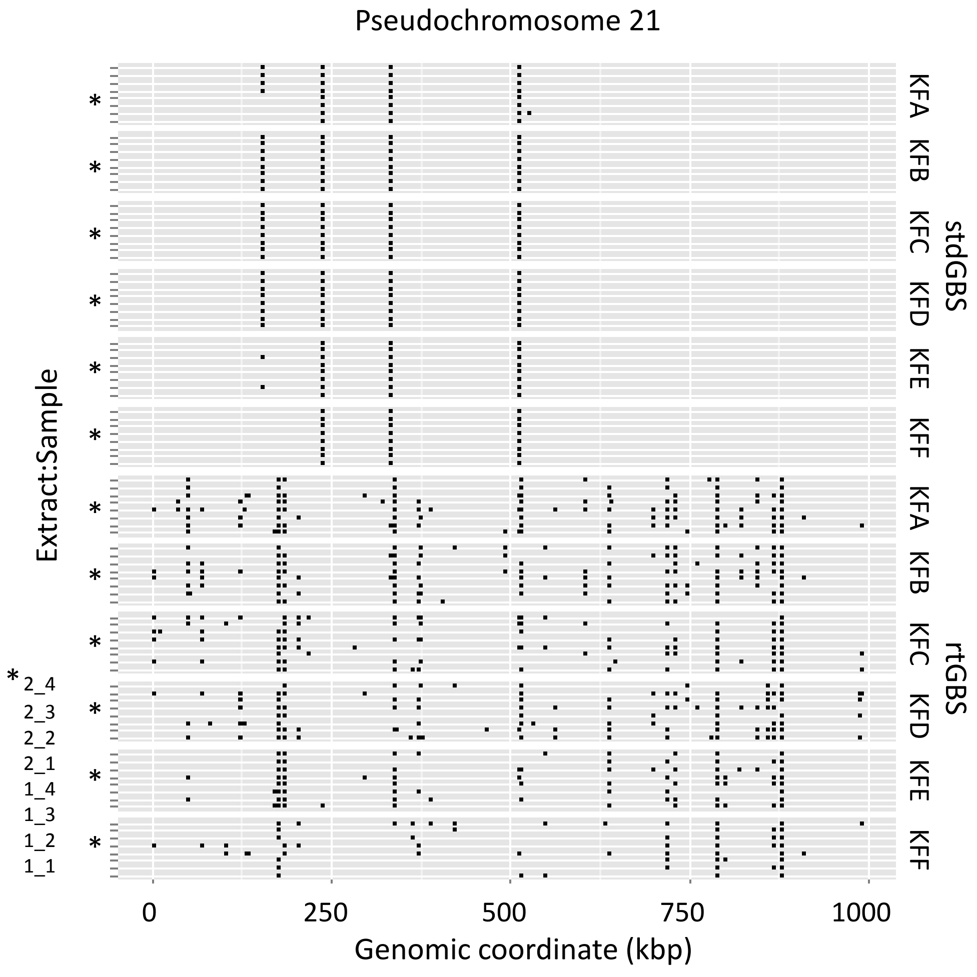

Pseudochromosome 21
KFA
KFB
KFC
KFD
KFE
KFF
stdGBS
KFA
KFB
KFC
KFD
KFE
KFF
rtGBS
Extract:Sample
*
2_4
2_3
2_2
2_1
1_4
1_3
1_2
1_1
0
250
500
750
1000
Genomic coordinate (kbp)

Pseudochromosome 22

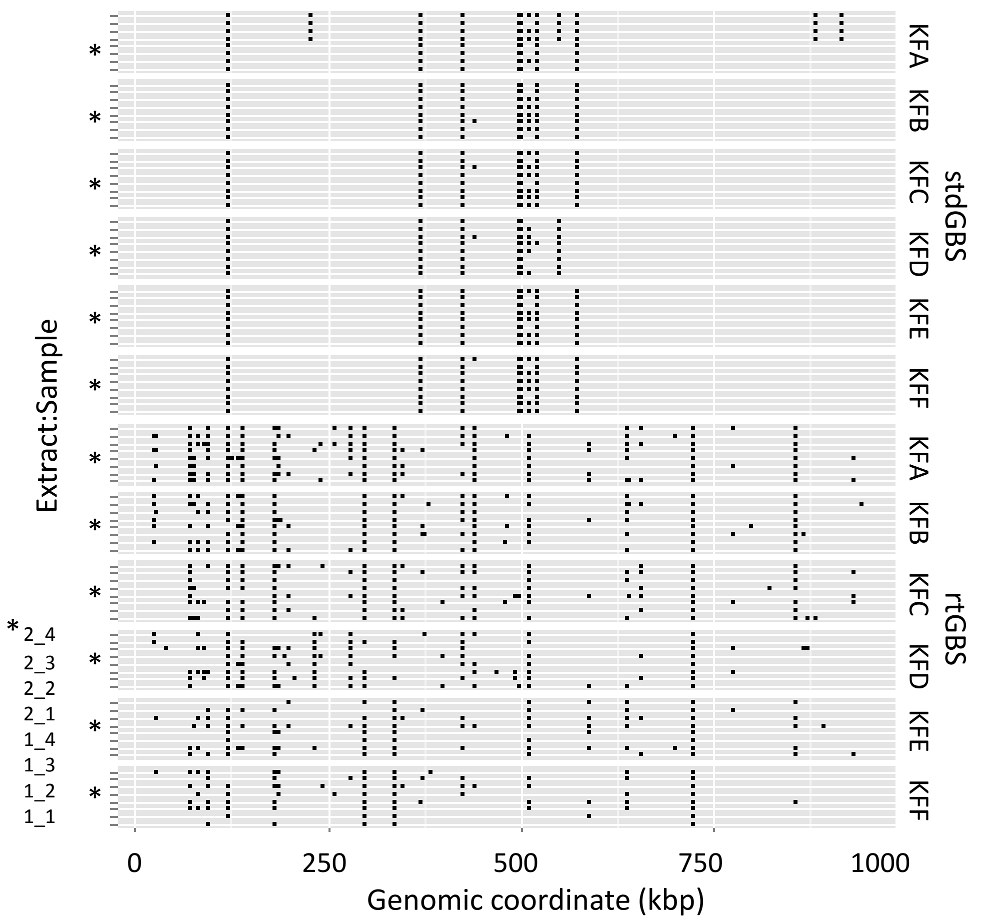

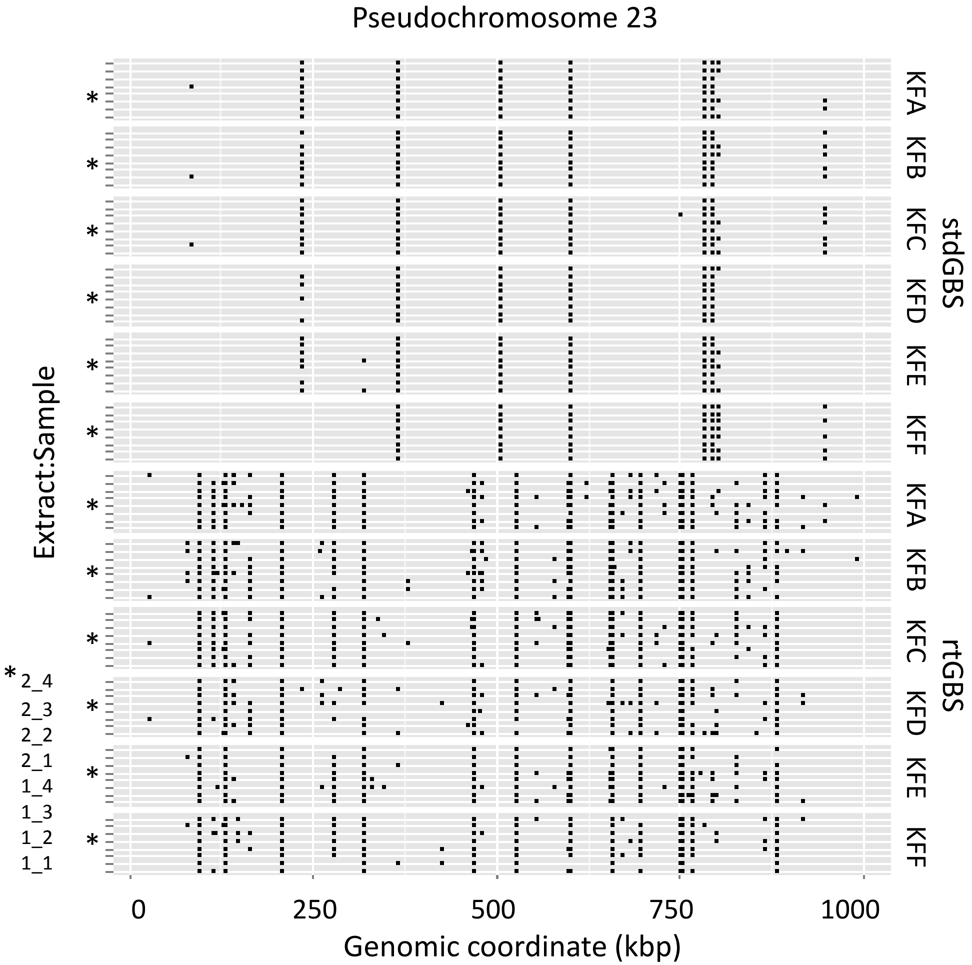

Pseudochromosome 23
KFA
KFB
KFC
KFD
KFE
KFF
stdGBS
KFA
KFB
KFC
KFD
KFE
KFF
rtGBS
Extract:Sample
2_4
2_3
2_2
2_1
1_4
1_3
1_2
1_1
0
250
500
750
1000
Genomic coordinate (kbp)

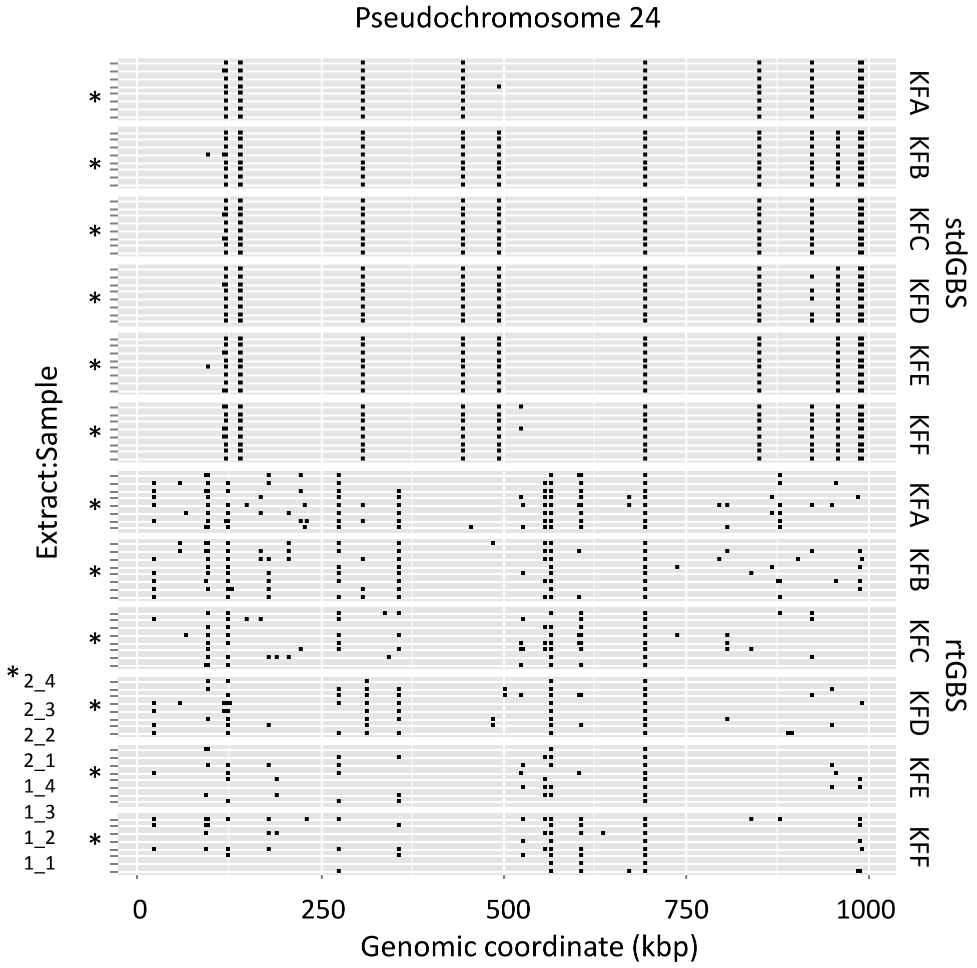

Pseudochromosome 24
Extract:Sample
2_4
2_3
2_2
2_1
1_4
1_3
1_2
1_1
0
250
500
750
1000
Genomic coordinate (kbp)
KFA
KFB
KFC
KFD
KFE
KFF
stdGBS
rtGBS

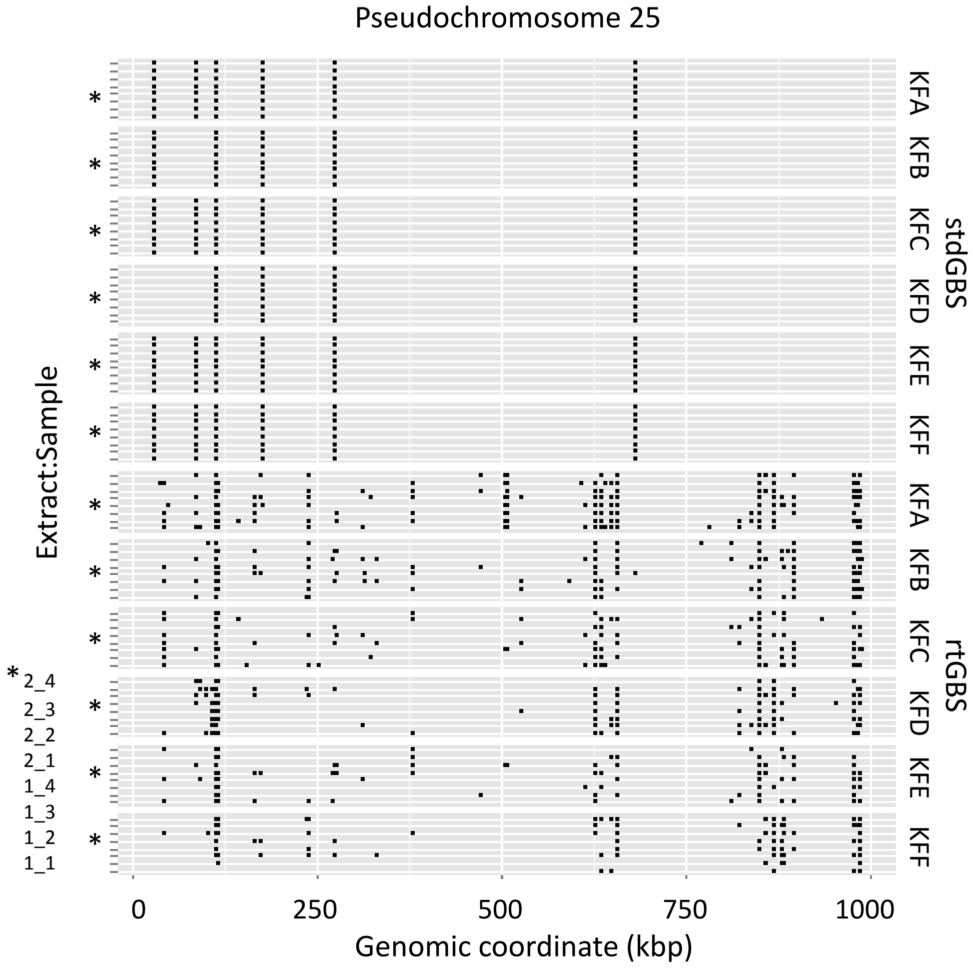

Pseudochromosome 25
KFA
KFB
KFC
KFD
KFE
KFF
stdGBS
KFA
KFB
KFC
KFD
KFE
KFF
rtGBS
Extract:Sample
*
2_4
2_3
2_2
2_1
1_4
1_3
1_2
1_1
0
250
500
750
1000
Genomic coordinate (kbp)

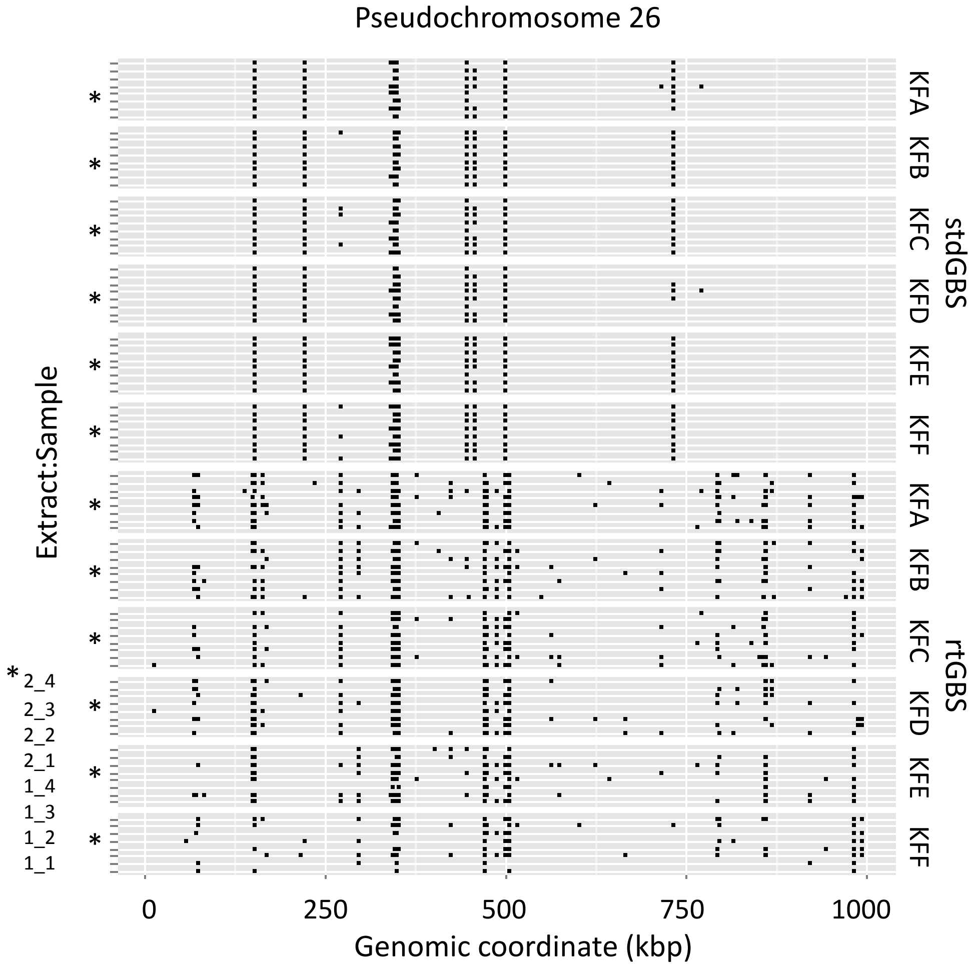

Pseudochromosome 26
Extract:Sample
stdGBS
rtGBS
KFA
KFB
KFC
KFD
KFE
KFF
KFA
KFB
KFC
KFD
KFE
KFF
*2_4
2_3
2_2
2_1
1_4
1_3
1_2
1_1
0
250
500
750
1000
Genomic coordinate (kbp)

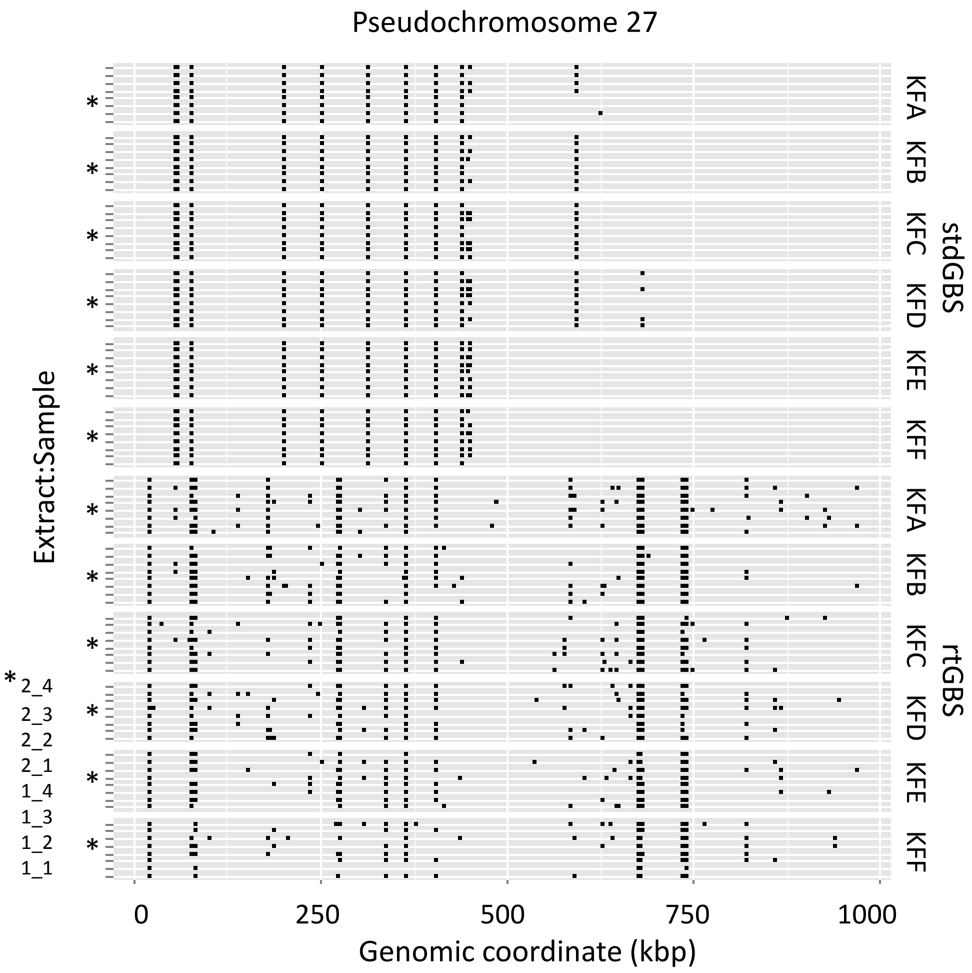

Pseudochromosome 27
Extract:Sample
stdGBS
rtGBS
KFA
KFB
KFC
KFD
KFE
KFF
2_4
2_3
2_2
2_1
1_4
1_3
1_2
1_1
0
250
500
750
1000
Genomic coordinate (kbp)

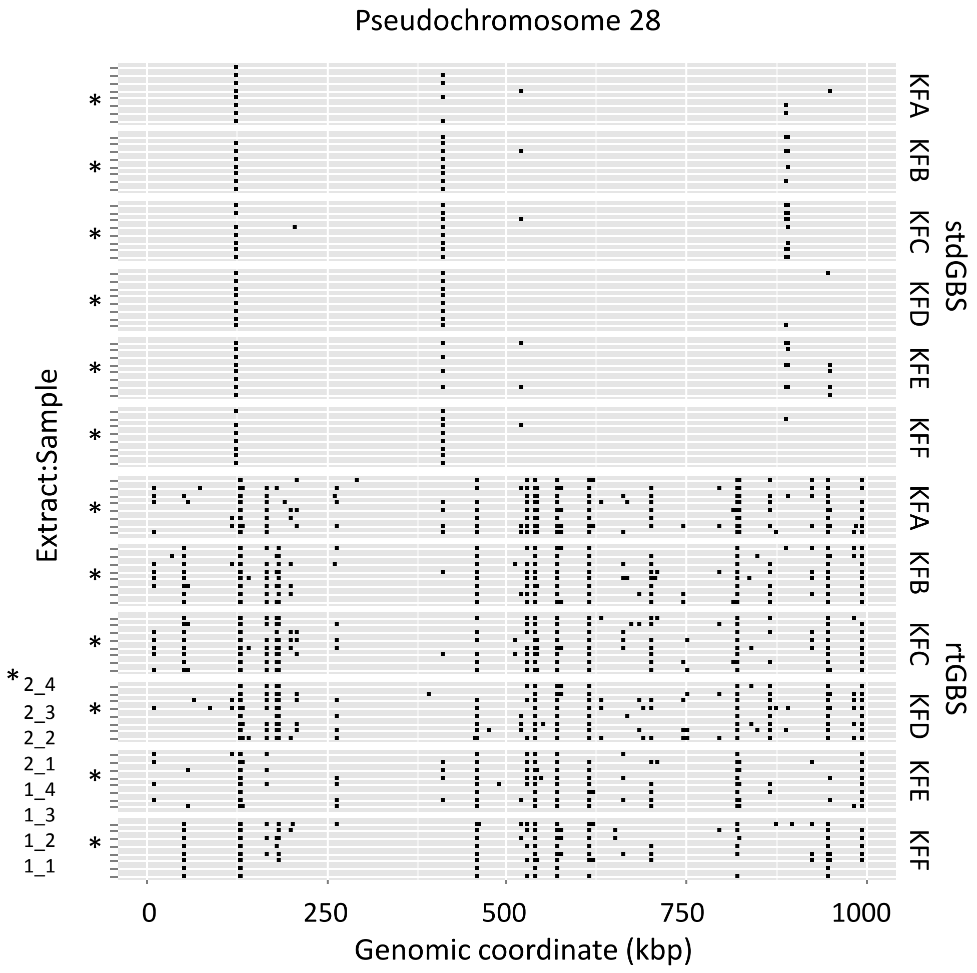
Pseudochromosome 28
Extract:Sample
stdGBS
rtGBS
KFA
KFB
KFC
KFD
KFE
KFF
KFA
KFB
KFC
KFD
KFE
KFF
*
2_4
2_3
2_2
2_1
1_4
1_3
1_2
1_1
0
250
500
750
1000
Genomic coordinate (kbp)

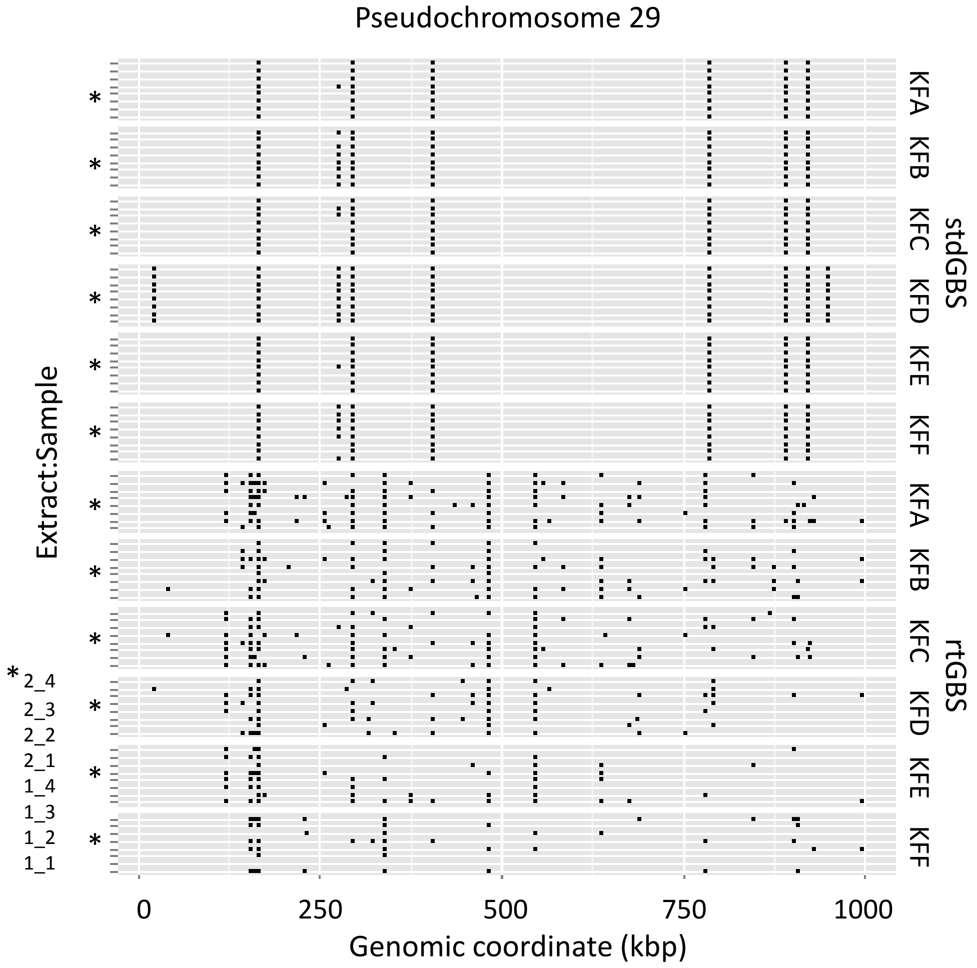
Pseudochromosome 29
Extract:Sample
KFA
KFB
KFC
KFD
KFE
KFF
stdGBS
KFA
KFB
KFC
KFD
KFE
KFF
rtGBS
2_4
2_3
2_2
2_1
1_4
1_3
1_2
1_1
0
250
500
750
1000
Genomic coordinate (kbp)

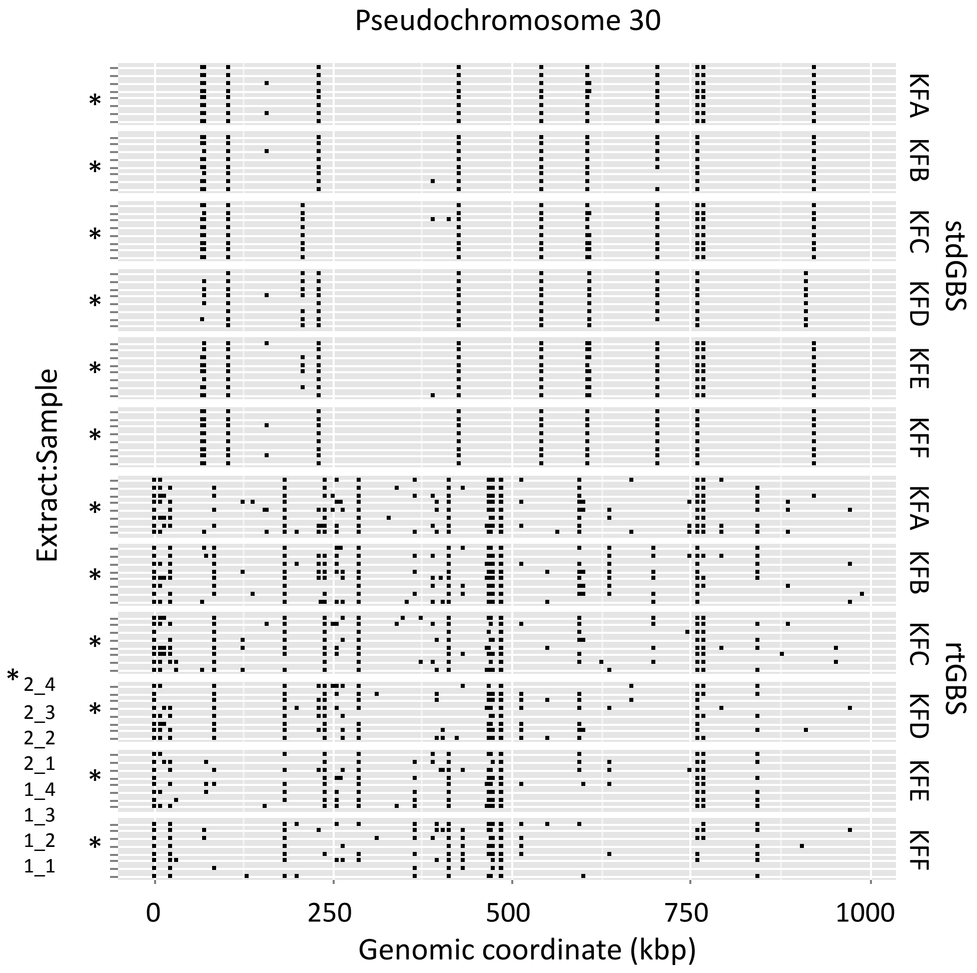

Pseudochromosome 30
Extract:Sample
stdGBS
rtGBS
KFA
KFB
KFC
KFD
KFE
KFF
KFA
KFB
KFC
KFD
KFE
KFF
2_4
2_3
2_2
2_1
1_4
1_3
1_2
1_1
0
250
500
750
1000
Genomic coordinate (kbp)
