# Supplementary material for: Random Tagging Genotyping by Sequencing (rtGBS), an Unbiased Approach to Locate Restriction Enzyme Sites across the Target Genome
Source: PLoS One. 2015 Dec 3;10(12):e0143193. doi: 10.1371/journal.pone.0143193 (PMC4669186; doi:10.1371/journal.pone.0143193)
Supplement: S1 Text — Further details of the modeling, the parameter estimates (presented on natural log scale) between the two models discussed in Table 2. (PDF) [file pone.0143193.s002.pdf]

Analysis of # BAMH1 cut sites detected under two different library preparation methods in GBS, i.e. Standard (sGBS) and Radom-tagging (rtGBS)

The data

Six kiwifruit plant biological replicates were each independently extracted twice, and then each extract sampled 4 times giving 8 laboratory replicates which formed a total of 48 experimental units for the study. The experimental units were each subjected to two different treatment methods (sGBS and rtGBS) generating 96 libraries for sequencing. The two treatment levels (sGBS and rtGBS) were therefore paired within each experimental unit. The 96 libraries were finally subjected to two independent amplifications which were run on separate lanes of the machine. Any systematic differences between lanes and amplifications were therefore confounded in the experimental design and this is labelled as a single factor 'Lane' with two levels. The data structure therefore comprised of 2 methods x 6 plants x 2 extracts x 4 samples x 2 lanes. It is noted here that the 48 experimental units constituted a nested structure i.e. plant, extract (plant) and aliquot (plant, extract). Each nested factor should be ideally treated as a random effect. The method (s or rt) and lane must be treated as fixed effects, but their interaction could be random. Any interactions of experimental unit factors with method may be of interest and should also be considered as a random effect. Initial analysis suggested that there were very small differences between extract and aliquot levels. Hence, for the purpose of this analysis these were collapsed to a single factor. The response variable of interest y_{ijkm} was defined as the number of BAMH1 cut sites estimated from reads mapped on to HongYang genome in the flow cell that had a library prepared from plant *i*, sample (extract x aliquot) *j* and then subjected to method *k* and sequenced in lane *m*.

Objectives of the statistical analysis

1. To ascertain if rtGBS increased the # BAMH1 cut sites compared to the sGBS method.
2. To quantify the variability in BAMH1 site count in relation to experimental unit factors.

The model specification

We specified two alternative statistical models for the analysis. The negative binomial distribution is commonly used to model count data when over-dispersion is present and the Poison approximation is not adequate, hence in the first model assumed,

$$\mathrm{y}_{\mathrm{ijkm}} \sim \mathrm{NB}(\mu_{\mathrm{ijkm}}, \sigma^2_{\mathrm{ijkm}}) \qquad (1)$$

which can be fitted as a generalised linear mixed model (GLMM) with the random and fixed effect predictors described earlier. The GLMMs are complex and difficult to fit and pseudo-likelihood methods are used for optimisation. The second model log transformed the counts data and assumed a normal distribution,

$$\log(\mathrm{y}_{\mathrm{ijkm}}) \sim \mathrm{N}(\mu_{\mathrm{ijkm}}, \sigma^2_{\mathrm{ijkm}}) \qquad (2)$$

which can be fitted as the simpler linear mixed model (LMM). Given the distribution assumptions, the linear model with predictors of the response can be specified as:

$$\log_e(\mu_{ijkm}), E[\log_e(y_{ijkm})] = \mu + P_i + S_j + m_k + l_m + Pm_{ik} \qquad (3)$$

where the plant $P_i \sim N(0, \sigma^2_P)$ and sample effect $S_j \sim N(0, \sigma^2_S)$ were both specified as random effects and captured the biological and laboratory variability respectively among the library experimental units. The method m_k and l_m in model (3) were considered fixed effects. Note the two terms on the left side of Eqn. (3) are the linear predictor for model (1) and (2) respectively.

The GLMM and LMM described as such were fitted to the data using the GLIMMIX and MIXED procedures in SAS® statistical software (SAS Institute Inc. 2013).

Results

Table 1 Estimates of fixed effect means and variance components of random effects obtained by fitting two alternative models (GLMM and LMM) to the number of BAMH1 cut sites collected on 48 experiment units x 2 methods x 2 lanes (and amplification). All estimates and their SEs are on the natural logarithm scale.

Effect		Estimate	
		GLMM	LMM
Fixed			
Method:	random-tagging	10.37 ±0.052	10.36 ± 0.055
	standard	9.83 ± 0.052	9.82 ± 0.055
Lane	1	10.12 ± 0.038	10.11 ± 0.040
	2	10.08 ± 0.038	10.07 ± 0.040
Random			
Plant		0.0	0.0
Sample x Extract (Plant)		0.0008 ± 0.0008	0.0009 ± 0.0009
Plant x Method		0.0157 ± 0.0074	0.0172 ± 0.0080
Residual		0.0106 ± 0.0013	0.0112 ± 0.0014

Table 2 Estimates of predicted Plant x Method means (on natural logarithm scale)

Method	Plant						
	A	B	C	D	E	F	SE
rtGBS	10.52	10.46	10.41	10.34	10.32	10.13	0.027
sGBS	9.87	9.85	9.86	9.62	9.82	9.94	0.027
Change in count	92.0%	85.2%	72.9%	105.5%	64.8%	20.9%	

Fig 1.1 Plots of conditional residuals of the negative binomial generalised linear mixed model fitted to BAMH1 cut site count data

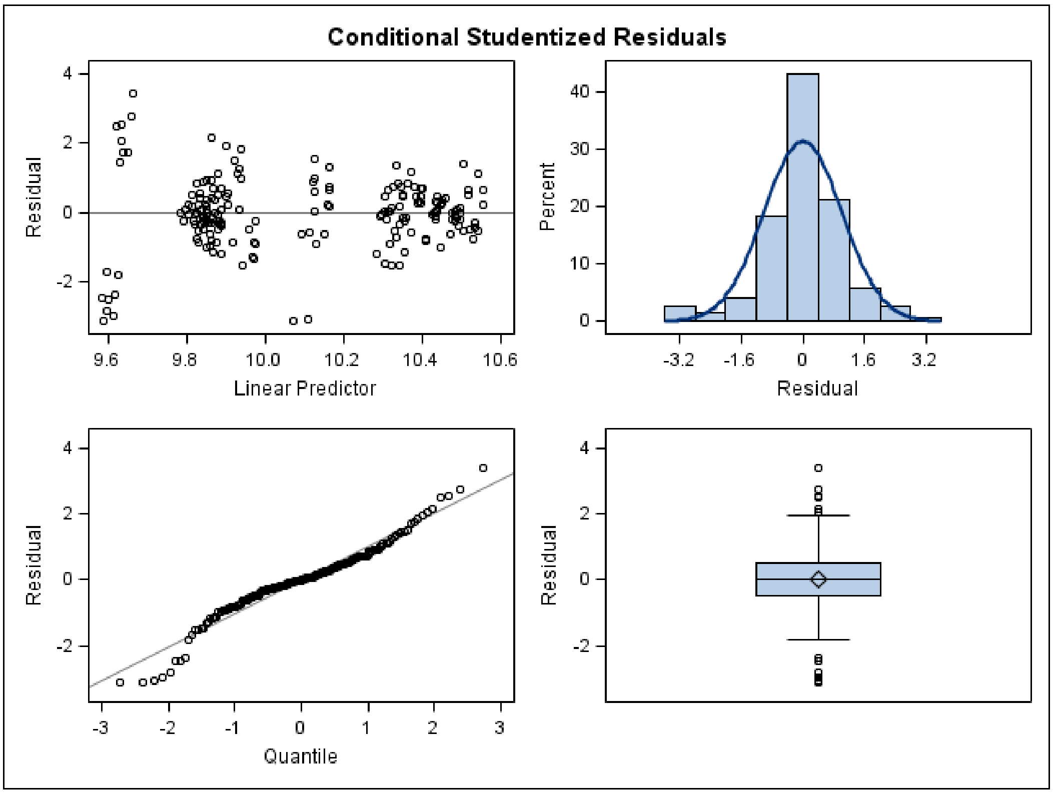

Fig 1.2. Plots of conditional residuals of the linear mixed model fitted to $\log_e$ (count)

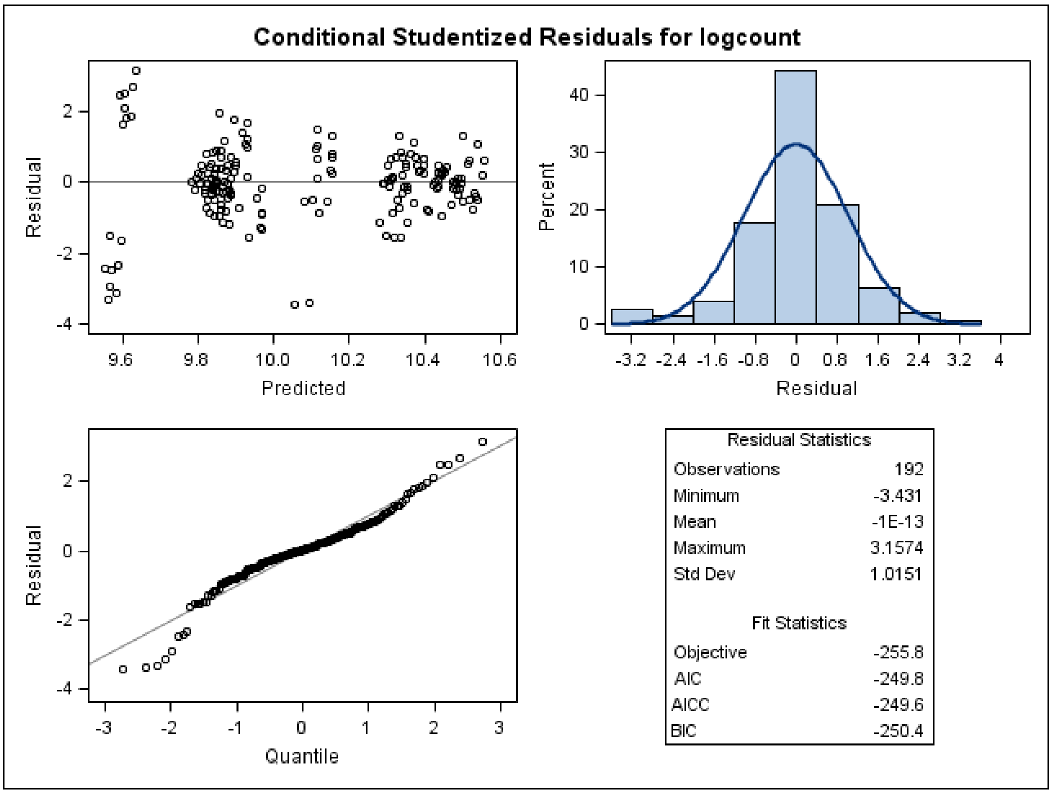

Comparing the cumulative distributions of the BAMH1 cut site count for the two methods

Fig 2. Empirical cumulative distribution function of BAMH1 cut site count for the two treatment methods

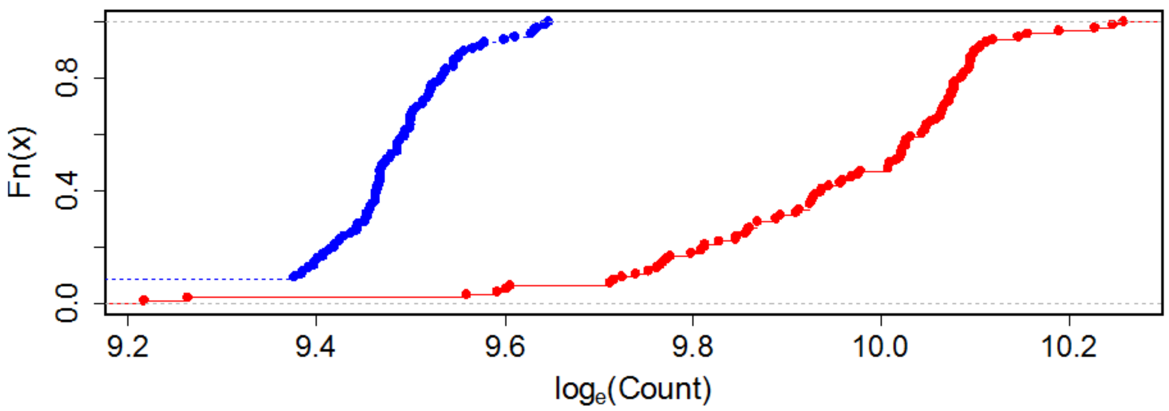

The shift in distribution as indicated by the two-sample Kolmogorov-Smirnov test was highly significant (*p*-value < 2.2E-16).
